# A non-ACE2 competing human single-domain antibody confers broad neutralization against SARS-CoV-2 and circulating variants

**DOI:** 10.1038/s41392-021-00810-1

**Published:** 2021-11-03

**Authors:** Zhenlin Yang, Yulu Wang, Yujia Jin, Yuanfei Zhu, Yanling Wu, Cheng Li, Yu Kong, Wenping Song, Xiaolong Tian, Wuqiang Zhan, Ailing Huang, Shanshan Zhou, Shuai Xia, Xiaoxu Tian, Chao Peng, Cuicui Chen, Yibing Shi, Gaowei Hu, Shujuan Du, Yuyan Wang, Youhua Xie, Shibo Jiang, Lu Lu, Lei Sun, Yuanlin Song, Tianlei Ying

**Affiliations:** 1 Department of Pulmonary Medicine, Zhongshan Hospital, Fudan University, Shanghai, 200032 China; 2 Shanghai Engineering Research Center for Synthetic Immunology, Shanghai, 200032 China; 3 Shanghai Key Laboratory of Lung Inflammation and Injury, Shanghai, 200032 China; 4 MOE/NHC Key Laboratory of Medical Molecular Virology, Shanghai Institute of Infectious Disease and Biosecurity, School of Basic Medical Sciences, Shanghai Medical College, Fudan University, Shanghai, 200032 China; 5 The Fifth People’s Hospital of Shanghai, Fudan University and Shanghai Key Laboratory of Medical Epigenetics, Institutes of Biomedical Sciences, Fudan University, Shanghai, 200032 China; 6 National Facility for Protein Science in Shanghai, Zhangjiang Lab, Shanghai Advanced Research Institute, Chinese Academy of Science, Shanghai, 201210 China; 7 Department of Pulmonary Medicine, Shanghai Respiratory Research Institute, Shanghai, 200032 China

**Keywords:** Infectious diseases, Structural biology

## Abstract

The current COVID-19 pandemic has heavily burdened the global public health system and may keep simmering for years. The frequent emergence of immune escape variants have spurred the search for prophylactic vaccines and therapeutic antibodies that confer broad protection against SARS-CoV-2 variants. Here we show that the bivalency of an affinity maturated fully human single-domain antibody (n3113.1-Fc) exhibits exquisite neutralizing potency against SARS-CoV-2 pseudovirus, and confers effective prophylactic and therapeutic protection against authentic SARS-CoV-2 in the host cell receptor angiotensin-converting enzyme 2 (ACE2) humanized mice. The crystal structure of n3113 in complex with the receptor-binding domain (RBD) of SARS-CoV-2, combined with the cryo-EM structures of n3113 and spike ecto-domain, reveals that n3113 binds to the side surface of up-state RBD with no competition with ACE2. The binding of n3113 to this novel epitope stabilizes spike in up-state conformations but inhibits SARS-CoV-2 S mediated membrane fusion, expanding our recognition of neutralization by antibodies against SARS-CoV-2. Binding assay and pseudovirus neutralization assay show no evasion of recently prevalent SARS-CoV-2 lineages, including Alpha (B.1.1.7), Beta (B.1.351), Gamma (P.1), and Delta (B.1.617.2) for n3113.1-Fc with Y58L mutation, demonstrating the potential of n3113.1-Fc (Y58L) as a promising candidate for clinical development to treat COVID-19.

## Introduction

The novel coronavirus disease 2019 (COVID-19), caused by severe acute respiratory syndrome coronavirus 2 (SARS-CoV-2), has led to unprecedented damage to global health and the economy.^1–3^ The COVID-19 symptoms range from mild to severe and even fatal, typically including fever, cough, polypnea, and pneumonia.^4,5^ SARS-CoV-2 has been considered as the third coronavirus causing a major outbreak following SARS-CoV and Middle East respiratory syndrome coronavirus (MERS-CoV), all of which belong to the betacoronavirus genus.^6,7^

The entry of SARS-CoV-2 into a human cell is initiated by the recognition of the human membrane protein angiotensin-converting enzyme 2 (ACE2) by the spike (S) glycoprotein on the viral surface.^8,9^ S protein exists as a homotrimer with each protomer consisting of an N-terminal S1 subunit and a C-terminal S2 subunit, cleaved by host proteases (such as furin) during viral formation.^10^ Upon binding to ACE2, S protein is further cleaved by serine protease TMPRSS2 at an S2’ site for priming.^10^ From its metastable prefusion conformation, the S protein undergoes dramatic conformational changes to form a highly stable post-fusion conformation, resulting in shedding of the S1 subunit and virus-host membrane fusion mediated by the S2 subunit.^9^ The S1 subunit comprises an N terminal domain (NTD), a receptor-binding domain (RBD), and two subdomains (SD1 and SD2). Demonstrated by cryo-EM structures, the RBD in the S1 subunit is highly dynamic and adopts ‘up’ (or ‘open’) and ‘down’ (or ‘close’) states, where ‘up’ corresponds to receptor-accessible and ‘down’ corresponds to a receptor-inaccessible state.^8,9^ It was also reported that the SARS-CoV-2 RBD is less exposed than that of SARS-CoV, potentially contributing to immune surveillance evasion and the widespread of the virus potentially.^11^

Recently, inactivated vaccines,^12–14^ mRNA vaccines^15,16^ and vector-based vaccines^17–19^ have been approved as prophylactic medicines to prevent COVID-19. In addition, recombinant protein subunits^20^ and therapeutic antibodies^21–23^ are under development as treatments. Hundreds of neutralizing antibodies targeting diverse immunogenic epitopes, especially on RBD or NTD, have been reported.^24–28^ However, the high production costs of monoclonal antibodies restrain their broad use as a treatment for respiratory infection. Small fragments of antibodies with ultra-high potency are arising to be promising therapeutics.^29,30^ In addition, with the emerging prevalence of SARS-CoV-2 mutant strains constantly challenging the efficacy of current treatments, it is imperative to investigate the neutralization mechanism for different types of antibodies, providing the basis for the rational design of antibody combinations with synergistic effect and avoiding immune escape. Here we report the development of a human single-domain antibody with high neutralizing potency against SARS-CoV-2 and its prevalent variants. The antibody recognizes the SARS-CoV-2 spike with a novel binding mode, presented as an entirely open conformation of the spike.

## Results

### Variants of n3113 neutralize SARS-CoV-2 pseudovirus with high potency

**Fig. 1:**
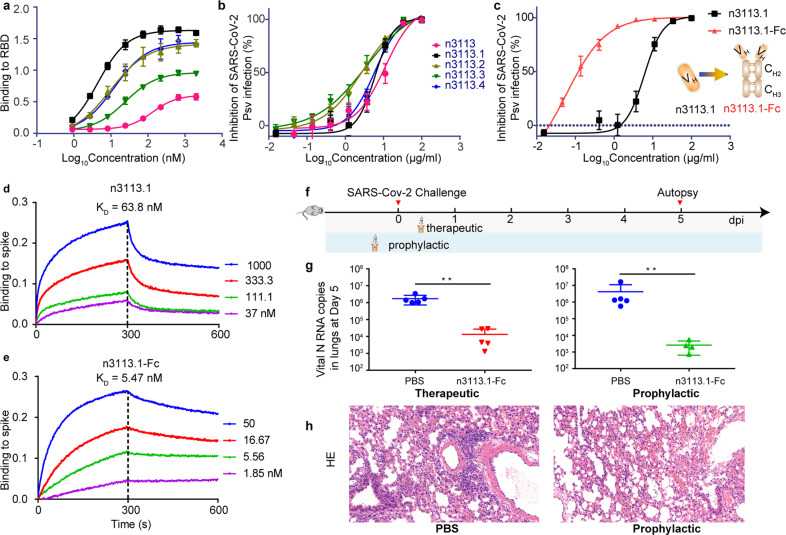
Human single-domain antibody shows therapeutic value against SARS-CoV-2. **a** Binding affinity of n3113 and its variants with SARS-CoV-2 RBD. The mean ± SD from three independent experiments is shown. **b** Neutralization of SARS-CoV-2 S pseudovirus by n3113 and its variants. The mean ± SD from three independent experiments is shown. **c** Schematic representation of the generation of n3113.1-Fc and in vitro SARS-CoV-2 S pseudovirus neutralizing activity of n3113.1 and n3113.1-Fc. Neutralizing activities are presented as mean ± SD from three independent experiments. **d**–**e** Binding kinetic of n3113.1 (**d**) and n3113.1-Fc (**e**) with immobilized prefusion S ectodomain, measured using bio-layer interferometry (BLI). Dissociation constant (K_D_) values for n3113.1 and n3113.1-Fc were obtained using a 1:1 binding model and 1:2 bivalent model, respectively. The experiments were performed in duplicate with similar results and a representative experiment is shown. **f** Schematic diagram of n3113.1-Fc treatment in SARS-CoV-2-susceptible mice. **g** Viral N RNA copies in the therapeutic group and the prophylactic group in the lungs were measured 5 dpi by qRT-PCR. *P* value was estimated by unpaired *t* test (**P < 0.01). **h** Histopathological characterization of the lungs of SARS-CoV-2 infected mice received prophylactic treatment of PBS or n3113.1-Fc

Previously, we isolated a fully human single-domain antibody, namely n3113, from a rapid and versatile antibody discovery platform.^31^ The antibody showed moderate SARS-CoV-2 S1 binding affinity and neutralization activity for HIV-1 based SARS-CoV-2 pseudovirus (half-maximal inhibitory concentration (IC_50_) of 18.9 μg/ml).^31^ To improve the binding affinity of n3113 with SARS-CoV-2 RBD, we introduced diversity into n3113 through error-prone PCR and generated a phage display library with an estimated diversity of 6 × 10^9^ (Supplementary Fig. 1a). Through polyclonal and monoclonal ELISA selection, 4 variants, namely n3113.1-4, showed one- to two-digital nano-mole affinity with RBD, and were subsequently screened and sequenced (Fig. 1a and supplementary Table 1). Compared to the pristine n3113 (118.9 nM), the binding affinity of the mutants with SARS-CoV-2 RBD was improved by 4 (26.6 nM, n3113.3) to 19 (6.4 nM, 3113.1) folds. The mutants were mildly more potent in neutralizing SARS-CoV-2 S pseudovirus than n3113 (IC_50_ between 2.8-6.0 μg/ml for n3113.1-4) (Fig. 1b and supplementary Table 1). We prioritized n3113.1 because of its relatively higher yield during purification. To further improve the neutralizing effect of n3113.1, we generated a bivalent variant of n3113.1 by fusing n3113.1 to the Fc domain of human IgG1 (n3113.1-Fc, Fig. 1c). This bivalency type of n3113.1 enhanced the SARS-CoV-2 S pseudovirus-neutralizing capacity dramatically by 2 orders of magnitude (IC_50_ of 6 μg/ml for n3113.1 and 0.06 μg/ml for n3113.1-Fc, Fig. 1c). Bio-layer interferometry (BLI) measurement of binding to immobilized SARS-CoV-2 spike showed that the bivalency was able to combat with the high dissociation rate of the monomer, resulting in a 12-fold increase of apparent affinity (dissociation constant, K_D_ of 63.8 nM for n3113.1 and 5.47 nM for n3113.1-Fc, Fig. 1d, e). The much higher activity of Fc-fused n3113.1 compared with n3113.1 (Fig. 1b) also indicated a critical role of antibody-induced avidity in neutralizing SARS-CoV-2.

### Protective efficacy of n3113.1-Fc in SARS-CoV-2-susceptible mice

In an authentic SARS-CoV-2 (nCoV-SH01)-infected human ACE2 (hACE2)-transgenic mouse model (1 × 10^5^ PFU virus per mouse), an intraperitoneal injection of n3113.1-Fc at a dose of 40 mg/kg at 2 h post intranasal injection of SARS-CoV-2 significantly reduced viral copies in the lung by 100 folds (Fig. 1 f, g). A more prominent effect (100−1,000 folds) was observed when n3113.1-Fc was administrated in a prophylactic mode, i.e., 2 h before the viral infection. Both prophylactic and therapeutic treatments of n3113.1-Fc protected the hACE2 mice from greater weight loss than control animals but the difference did not achieve statistical significance (Supplementary Fig. 2). The prophylactic treatment with n3113.1-Fc alleviated the lung injury, including inflammatory cell infiltration and alveolar septal thickening caused by SARS-CoV-2 infection, as revealed by histopathological examination (Fig. 1h). N3113.1-Fc has comparable protective efficacy in vivo with B38^32^ and H014^33^, and is a potential therapeutic treatment for COVID-19.

### Structural basis of n3113 binding modes at RBD and S trimer

**Fig. 2:**
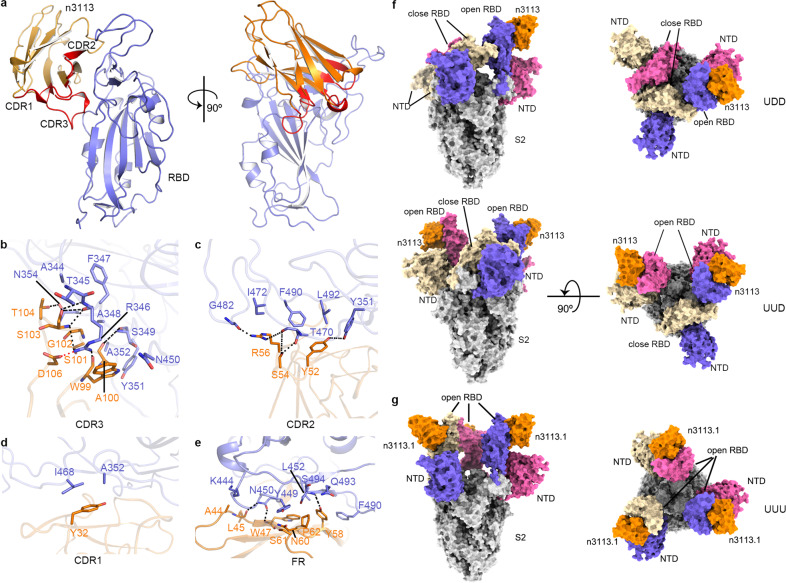
Structural features of n3113 and n3113.1 in complex with RBD and spike ectodomain. **a**–**e** Crystal structure of RBD-n3113. **a** Overall structure of RBD-n3113. The RBD is shown in a blue cartoon. N3113 is presented in an orange cartoon with its three CDRs highlighted in red. **b**–**e** Detailed interactions between CDR3 (**b**), CDR2 (**c**), CDR1 (**d**), and FR (**e**) of n3113 and RBD. The residues involved in binding are shown in blue (n3113) and orange (RBD) sticks. Salt bridge and hydrogen bonds are shown as red and black dashed lines, respectively. **f**, **g** Cryo-EM structures of spike ectodomain with n3113 (**f**) and n3113.1 (**g**) Two perpendicular views of UDD-, UUD- and UUU-state complex are shown as surface. S2 subunit of S trimer is rendered in grey. The S1 (NTD and RBD) subdomains from each monomer are colored in blue, pink, and brown, respectively. N3113 and n3113.1 are colored in orange

To better understand the molecular feature of the interaction pattern between RBD and n3113 and to provide insight into the neutralization mechanism of n3113, we determined the crystal structure of n3113 in complex with RBD at a resolution of 2.27 Å (Fig. 2a and supplementary Fig. 1b,c and Table 2).

The single-domain antibody n3113 is composed of antiparallel β-strands with its main complementarity determining region (CDR3, 12 amino acids) exhibiting a convex-shaped paratope^34^ and bent over towards RBD (Fig. 2a). Of 940 Å^2^ buried surface area (BSA), CDR3 accounts for 375 Å^2^. 7 (W99-T104 and D106) of the 12 residues in CDR3 participate in the interaction with RBD, forming 7 pairs of hydrogen bonds and 1 pair of a salt bridge (Fig. 2b, supplementary Table 3). The R346 locating in the α1–β1 loop of RBD plays a pivotal role in mediating the binding, as it is engaged in five pairs of hydrogen bonds (with S103 and W99 of n3113) and a salt bridge (protonated guanidyl side chain of RBD-R346 with the carboxyl side chain of n3113-D106). In addition, R346 is involved in a π-cation interaction with W99 of n3113 (Fig. 2b and supplementary Table 3). The critical role of R346 has been proven by the mutagenesis data as substitution of arginine to tryptophan, leucine or glutamate abolished the binding of RBD with n3113 (Supplementary Fig. 1d). In the RBD-n3113 structure, the side chain of R346 flips to almost the opposite direction when compared with the ACE2 bound structures (Supplementary Fig. 1e).^35,36^ Moreover, when complexed with the neutralization antibody 2F6 or B38 (Supplementary Fig. 1e),^37,32^ the side chain of RBD-R346 occupies different orientations, suggesting the flexibility of R346 in RBD upon binding to different counterparts.

The CDR2 of n3113 also forms an extensive hydrogen bond network with RBD, including four pairs of hydrogen bonds that involve Y52, S54, and R56 of n3113 and Y351, T470 and G482 of RBD (Fig. 2c, supplementary Table 3). Different from the intensive interactions between n3113 CDR2/CDR3 and RBD, the CDR1 of n3113 sits away from the RBD interface, and only mild hydrophobic interaction between Y32 of n3113 CDR1 and A352 and I468 of RBD is observed (Fig. 2d).

Surprisingly, apart from the three CDRs, the framework regions (FRs) 2 and 3 around n3113-CDR2 go parallel with the receptor-binding ridge and contribute 39% of the BSA with RBD (Fig. 2e). The backbone amide of W47 in FR2 of n3113 forms a hydrogen bond with the hydroxyl side chain of RBD-N450. Along with Y58, the N60-S62 in n3113 FR3 forms hydrophobic and hydrophilic interactions with Y449, L452, F490, Q493, and S494 of RBD, further strengthening the binding. Participants of FRs may be a compensation for the absence of light chain pairing in the interaction between n3113 and RBD.

In general, 10 of the 18 residues of RBD that are enrolled in the footprint of n3113 lack conservation between SARS-CoV and SARS-CoV-2 (Supplementary Fig. 3), rationalizing the lack of cross-reactivity observed for n3113.^31^

To further estimate the binding pose of n3113 on the SARS-CoV-2 trimeric spike, we determined the cryo-EM structure between a prefusion-stabilized ectodomain of the spike (Methods) and n3113. Cryo-EM characterization revealed two states of the complex, the UUD (up-up-down) and UDD (up-down-down)-states (Fig. 2f). The global map of UUD and UDD were determined at a resolution of 3.55 Å and 3.7 Å, respectively (Supplementary Fig. 4 and Table 4), in which coordinates from the S trimer and crystal structure of RBD-n3113 were fit by rigid-body docking. Unexpectedly, n3113 binds exclusively to the up-state RBD in the dynamic spike albeit its paratope is theoretically accessible in both up and down states. Superimposition of the crystal structure of RBD-n3113 to the down-state SARS-CoV-2 RBD in spike revealed that n3113 would clash with the glycan of N165 in adjacent NTD (Supplementary Fig. 1f), rationalizing its exclusive binding to up-state RBD. A previous research indicated that the mutation N165Q became more sensitive to mAb P2B-2F6,^38^ highlighting the removal of glycan modification in NTD for better recognition by the antibodies and the shielding by the glycan as self-defense of virus against the immune system.

Three-dimensional classification showed that 50.3% of the particles were at the UUD-state (Supplementary Fig. 4) while a predominant closed information of SARS-CoV-2 spike (54%) was reported when no antibody was added,^8^ indicating the propensity for up-state RBD upon incubation with n3113. A more significant phenomenon was observed when the spike was incubated with n3113.1, which showed a 30-fold enhancement of RBD binding affinity than n3113, as 60% of the particles were resolved as an up-up-up (UUU) state (Fig. 2g and Supplementary Fig. 5 and Table 4). The up-state-RBD preference of n3113 underscores the potential of n3113 as a prime candidate for combination treatment with other exposed receptor-binding subdomain-targeting monoclonal antibodies (mAbs) such as CB6, B38, C102, C105, CC12.1, CC12.3, COVA2-4, and REGN 10933 (Supplementary Fig. 6).

### N3113 inhibits viral-cell fusion with no competition with ACE2 for RBD binding

**Fig. 3:**
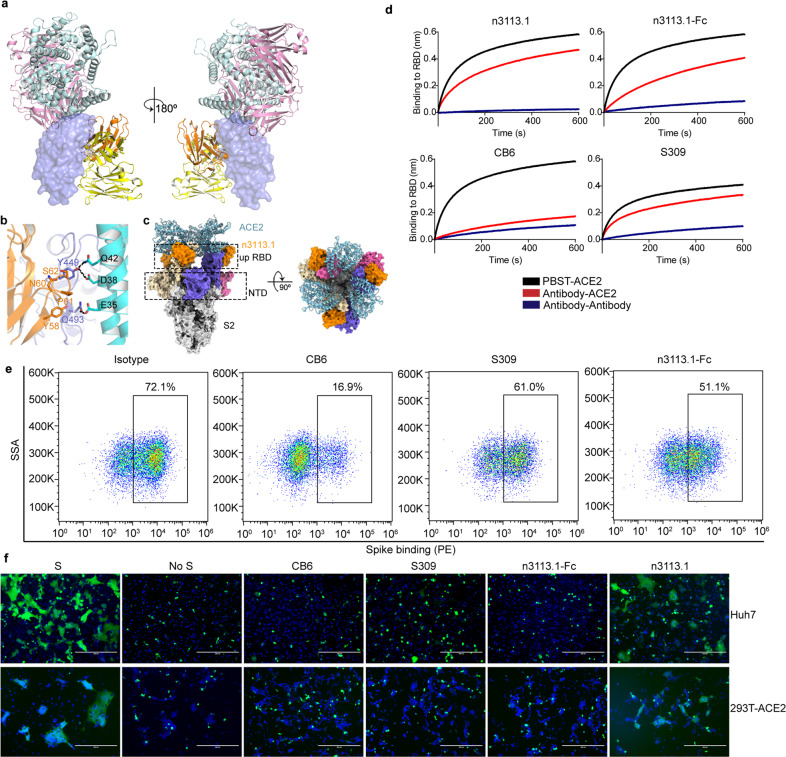
N3113 is a non-ACE2 competing antibody that inhibits SARS-CoV-2 S protein-mediated membrane fusion. **a** Structural depiction of n3113, ACE2 (PDB entry 6LZG), CB6 (PDB entry 7C01) and S309 (PDB entry 6WS6) binding to SARS-CoV-2 RBD. RBD is represented as a blue surface. ACE2, n3113, CB6 Fab, and S309 Fab fragments are shown as cartoon colored in cyan, orange, pink and yellow, respectively. **b** Binding mode comparison of Y449 and Q493 in SARS-CoV-2 RBD (blue) with ACE2 (cyan) and n3113 (orange). Sidechains of RBD-Y449, RBD-Q493, n3113-Y58, n3113-N60, n3113-P61, n3113-S62, ACE2-E35, ACE2-D38, and ACE2-Q42 are shown as sticks. The hydrogen bonds are represented in black dashed lines. **c** N3113 does not compete with ACE2 on binding to trimeric spike. Trimeric spike is represented in surface colored the same as in Fig. 2g. The superposed ACE2 is presented as a cyan cartoon. **d** Competition of n3113.1, n3113.1-Fc, CB6, and S309 with ACE2 for SARS-CoV-2 RBD binding were measured by BLI. Immobilized SARS-CoV-2 RBD were saturated with antibodies (1 µM n3113.1 or 100 nM n3113.1-Fc, CB6, S309), followed by incubation of the sensors with the corresponding antibody in the presence of (red) or without (blue) 200 nM soluble ACE2. As a control, the immobilized RBD was balanced in buffer and incubated with the equal molar of ACE2 (black). The grams show binding patterns after antibody saturation. **e** Analysis of S protein attachment to HEK293T-ACE2 cells. HEK293T-ACE2 cells were stained with 10 µg/mL his-tagged SARS-CoV-2 spike proteins pre-incubated with isotype IgG, CB6, or n3113.1-Fc. The percentage of binding was measured by anti-histag PE and analyzed by FACS. The experiments were performed twice with similar results and a representative figure was shown. **f** N3113 inhibits SARS-CoV-2 S protein-mediated membrane fusion. Membrane fusion was indicated by the formation of syncytium at 48 h after incubation. No antibody was added in the mock group (S). 10 μM n3113.1, n3113.1-Fc, S309, and CB6 were added individually to 293T-S-GFP cells before the incubation. 293 T cells transfected only with GFP was used as control (no S)

Superimpose of the crystal structure of RBD-n3113 and RBD-ACE2^36^ reveals no intrinsic footprint overlap (Fig. 3a). Even though both ACE2 and n3113 make contacts with Y449 and Q493 of RBD, the interactions seem to be compatible and do not interfere with each other (Fig. 3b), which is congruent with the BLI assay showing no competition between ACE2 and n3113 when binding to RBD.^31^ Docking of the crystal structure of SARS-CoV-2 RBD in complex with ACE2 onto the 3-up-state cryo-EM structure of spike-n3113.1 revealed that three molecules of ACE2 could be sterically accommodated in the UUU-state complex (Fig. 3c). We also carried out a competition assay of n3113.1 and other two antibodies, CB6^39^ and S309^40^ with ACE2 for SARS-CoV-2 RBD binding by BLI (Fig. 3a and d). The RBM-binding CB6 showed complete competition with ACE2 for binding to SARS-CoV-2 RBD. In contrast, n3113.1 and S309 were incapable of competing with ACE2 for RBD binding, in congruent with previous findings.^31,40^ However, we noticed that n3113.1-Fc displayed moderate competition, which may be caused by the steric hindrance between Fc or the other molecule of n3113.1 and ACE2. The BLI data were compatible with the binding results presented in the blocking assay by fluorescence-activated cell sorting (FACS) (Fig. 3e and Supplementary Fig. 7).

The entry of SARS-CoV-2 into host cell initiates with virus attachment to the surface of target cells engaging ACE2 as a cellular receptor, and is followed by the fusion of viral and cellular membranes facilitated by cellular protease-mediated S protein priming. To further ascertain the mechanism of SARS-CoV-2 inhibition by n3113 and S309, we performed a SARS-CoV-2 S protein-mediated cell-cell fusion assay^41,42^(Fig. 3f). 293 T cells transiently transfected with SARS-CoV-2 S protein and GFP were incubated with target Huh7 cells that endogenously express ACE2 or 293 T cells engineered to express ACE2 at their surface for 48 h. Large syncytium with multiple nuclei was observed in fused cells, due to the S protein-mediated membrane fusion. Pretreatment of effector 293T-S cells with 10 μM n3113.1 had moderate prevention for syncytia formation. However, n3113.1-Fc and S309 prevented the cell-cell fusion profoundly. The inhibition potency of these two antibodies is comparable with that of CB6. These data indicated that n3113.1 and S309 can block viral entry into healthy cells through inhibiting SARS-CoV-2 fusion with the target cells.

### N3113 retains sensitivity to the circulating SARS-CoV-2 variants

**Fig. 4:**
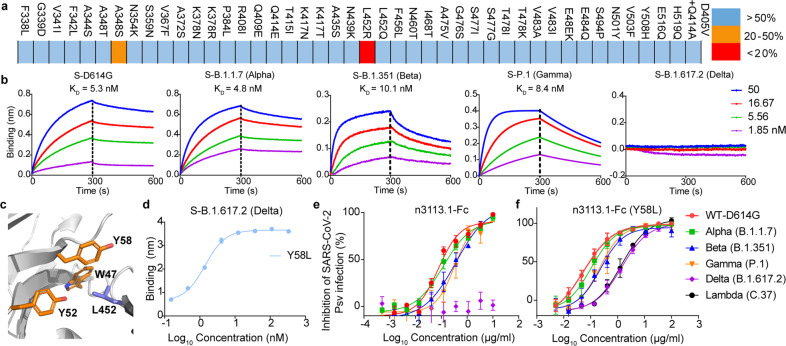
N3113.1 (Y58L) maintains susceptivity to circulating SARS-CoV-2 variants. **a** Binding affinity of n3113.1 with RBD variants. Colors represent the extent of binding affinity percentage relative to wild-type RBD: blue, over 50%; orange, 20% to 50%; red, less than 20%. **b** Binding kinetics of n3113.1-Fc to spike variants, as measured by BLI. K_D_ values for n3113.1-Fc were obtained using a 1:2 bivalent model. **c** Binding mode of RBD-L452 and n3113. RBD and n3113 are shown as cartoon, colored in blue and orange, respectively. RBD-L452 and residues on n3113 that form hydrophobic interactions are shown as sticks. **d** Binding of Y58L mutations based on n3113.1-Fc to S protein of Delta variant. The mean ± SD from two independent experiments is shown. **e** Neutralization profiles for n3113.1-Fc against viruses pseudotyped with the S protein of WT and four VOCs. Two independent experiments were performed in triplicate. **f** Neutralization profile of n3113.1-Fc (Y58L) against viruses pseudotyped with the S protein of WT, four VOCs, and one VOI. The mean ± SD from three independent experiments is shown

The S protein of SARS-CoV-2 constantly mutates.^38^ Multiple variants of SARS-CoV-2 have been reported, of which some are defined as variants of concern (VOCs), and several are considered variants of interests (VOIs).^43^ The newly emerging variants confer a higher level of resistance to convalescent and vaccine serum, as well as therapeutic mAbs, challenging the current therapy.^44–47^ We investigated several RBD variants within publicly available SARS-CoV-2 sequences in the Global Initiative on Sharing All Influenza Data (GISAID)^48^ and all of the individual RBD mutants (N501Y, E484K, E484Q, K417N, K417T, L452R, L452Q, T478K) found in dominant VOCs (B.1.1.7, Alpha; B.1.352, Beta; P.1, Gamma; B.1.617.2, Delta; B.1.427/B.1.429, Epsilon) for n3113.1 binding. These mutations are also shared by some VOIs – Zeta (P.2), Eta (B.1.525), Theta (P.3), and Kappa (B.1.617.1). N3113.1 binds to most of the mutations including the N501Y and E484K/Q which were reported to have increased binding potency of spike with ACE2 and appeared in several VOCs and VOIs, with similar affinity as WT RBD (Fig. 4a). However, the binding affinity of n3113 decreased by 20-50% for A348S and over 90% for L452R (Fig. 4a and supplementary Fig. 8a).

During the circulation of SARS-CoV-2, the notable mutation D614G became the first dominant variant and replaced the ancestral spike. The mutation increases viral infectivity through shifting the equilibrium to up conformation of RBD in the trimeric S protein.^49^ We generated a D614G mutation into the prefusion-stabilized spike for cryo-EM structure determination. The expressed S protein was immobilized and the dissociation constants (K_D_) of n3113.1-Fc with the mutant spike was tested by BLI assay. D614 locates at SD2 and is not involved in the binding epitope of n3113. In align with this, the binding affinity of n3113.1-Fc with D614G spike showed no difference with the wild-type spike (WT S) (5.5 nM for WT S and 5.3 nM for D614G S, Fig. 1e and Fig. 4b). We also tested the binding of n3113.1-Fc and spike variants of four VOCs, Alpha, Beta, Gamma, and Delta. N3113.1-Fc bound to the spike variants of Alpha, Beta, and Gamma with a comparable affinity of 4.8 nM, 10.1 nM, and 8.4 nM, respectively, but lost the binding ability to Delta spike that contained L452R mutation (Fig. 4b). In vitro HIV-1-based SARS-CoV-2 S pseudovirus neutralization assay presented the same result (Fig. 4e). N3113.1-Fc neutralized pseudoviruses loaded with a spike of WT-D614G and Alpha variant in indiscriminate potent (IC50 of 0.07 μg/ml and 0.12 μg/ml for WT-D614G and Alpha, respectively, Fig. 4e). N3113.1-Fc was fivefold less potent against both the Beta (0.37 μg/ml) and Gamma (0.33 μg/ml) variant relative to the parental viruses. However, the whole S mutation of B.1.617.2 endowed pseudovirus resistance to neutralization by n3113.1-Fc (Fig. 4e).

The high-resolution crystal structure of RBD and n3113 reveals that L452 locates on RBM and interacts with a hydrophobic cluster formed by W47, Y52, and Y58 of n3113 (Fig. 4c). The replacement of leucine with bulky arginine thus causes steric hindrance with W47, Y52, or Y58 and abolished the interaction, rationalizing observed escape. Interestingly, the glutamine substitution of leucine (L452Q) has increased the binding affinity of RBD to n3113.1 (Supplementary Fig. 8a). Whilst the over-long length of the side chain of arginine destroys the interaction, the intermediate length of the side chain of glutamine may strengthen the interaction between RBD and n3113.1. Based on these findings, we tried mutations of Y52, Y58, and W49 on n3113.1 to shorten the side chains and found a single Y58L mutation could recover the binding ability of n3113.1-Fc to Delta S (Fig. 4d). In alignment with this, the Y58L mutant on n3113.1-Fc reverted susceptivity to virus pseudotyped with Delta S (Fig. 4f). In addition, the mutations didn’t change the neutralizing potency to the parental virus (Fig. 4f).

The noticeable VOI - C.37 (Lambda) contained both F490S and L452Q mutations in its RBD region. Although n3113.1-Fc showed impaired binding (10-fold less than WT S) to the spike protein, the apparent binding affinity of n3113.1-Fc to F490S/L452Q double mutation containing RBD was determined at higher than 0.5 nM (Supplementary Fig. 8b) and n3113.1-Fc neutralized pseudoviruses loaded with Lambda S at 0.36 μg/ml (Supplementary Fig. 8c). The unperturbed binding potency of n3113-Fc (especially with Y58L mutation) to variant spikes and the robust in vivo protection of n3113-Fc in SARS-CoV-2-infected mice indicate that n3113.1-Fc (Y58L) is potentially effective in conferring the antigenic drift of present emerging variants.

## Discussion

Three-dimensional structures of antibodies in complex with SARS-CoV-2 RBD or trimeric S protein explicitly unravel three types of binding epitopes, ACE2-competing site, cryptic site inside the trimeric interface, and epitope outside the trimeric interface.^32,33^^,^^37,39,40,50–54^ N3113 and other reported antibodies, such as S309,^40^ C135,^54^ 47D11^55^ and Nb17,^56^ belong to the third class. The primary neutralization mechanism for ACE2-competing antibodies is defined as a blockade of spike-receptor interaction and the vast majority of highly potent antibodies occupy the receptor-binding motif (RBM) and have steric hindrance with ACE2, exemplified by P2B-2F6,^37^ CB6^39^ and BD23.^50^ Antibodies that bind to the cryptic site destruct the prefusion spike.^57^ Previously, we found a human mAb, CR3022, originating from the memory B cells of SARS survivors, bound potently with SARS-CoV-2 RBD.^58^ CR3022 recognizes a highly-conserved cryptic epitope buried in the trimeric interface^53^ and doesn’t compete with ACE2. Whilst CR3022 failed to neutralize SARS-CoV-2 in vitro, EY6A, sharing a similar footprint with CR3022, showed in vitro SARS-CoV-2 neutralizing ability. Cryo-EM images revealed that both CR3022^57^ and EY6A^52^ incubation destroyed the structural integrity of the spike.

Though the neutralizing mechanism of the antibodies that fall into the above two classes are well documented, it remains hitherto ambiguous for ACE2 non-competing antibodies that bind to the side surface of RBD, with the limited cognition that S309 potentially blocks viral entry and activates effector functions to clear infected cells.^40^ Discrepant from the glycopeptidic epitope of S309, C135, and 47D11, the binding site of n3113 doesn’t contain the oligosaccharide N343 and is much closer to the apex of spike protein (Supplementary Fig. 9). RBD in spike undergoes “up” and “down” conformational changes dynamically. Cryo-EM structures show that S309 and C135 recognize both the up and down RBD, while 47D11 binds specifically to the closed RBD.^55^ Taken the vertical line crossing N343 as coordinate line, we found that the epitope of n3113 locates at an upper and right site, close to the receptor-binding ridge of RBD. Oppositely, 47D11 binds to a left and lower site. Neutrally, S309 and C135 bind to both sides, locating at the middle region of RBD. The relative site compared with the N343 position may be the determinant for different binding modes of side-surface binding antibodies on dynamic RBD. Moreover, the sub-stoichiometric binding observed in the cryo-EM structure of n3113 and 47D11 may account for their lower binding potency.

It was well known that the spike-mediated fusion of viral and cellular membrane undergoes a cascade of preceding events. (1) Recognition of the virus by host cell through interaction between spike and ACE2. (2) Priming of a spike by host cell membrane proteases cleavage after the conformational rearrangement and exposure of S2’ site induced by S-ACE2 interaction. (3) Shedding of S1. (4) S2 mediated fusion of viral and cellular membrane. The procedures may happen simultaneously. Both n3113.1 and S309 inhibit cell-cell membrane fusion (Fig. 3e). The exact mechanism underlying the inhibition activity is hard to characterize due to the absence of competition between antibodies and ACE2.

HDX MS (hydrogen-deuterium exchange mass spectrometry) experiments revealed three regions in a spike that have a different rate of uptake of deuterated solvent upon n3113.1 binding (Supplementary Fig. 10). The lower exchange rate for 438-452 in RBD is coordinate with the protection of the epitope by n3113.1. A similar slower rate was observed in peptide 627-641, which belongs to the SD2 region of a spike. Although the peptide 627-641 is unresolved in the EM structure, the results indicated a stable conformational change of this part when bound to n3113.1. This region was designated as a 630 loop and proposed to modulate stabilization of premature S and the fusogenic structural rearrangement of S.^59,60^ On the other hand, the conformational heterogeneity of peptide 553-568 (SD1) was implied by faster uptake rate of deuterium. The above finding showed that SD1 and SD2, which were reported as hinges for RBD movement, was induced by n3113.1 to undergo dynamic changes. We assumed that the main function of n3113 might be to lock the RBD in the “open/up” orientation and refold the SDs to stabilize premature S, simultaneously preventing the structural arrangement in the S protein required to drive membrane fusion. However, considering that our understanding of SARS-CoV-2 entry and neutralization mechanisms is still evolving, the above hypothesis doesn’t rule out other neutralizing possibilities.^61,62^

Up-state of RBD is typically considered as necessary for activation by receptor binding. Thus, for the major instances, neutralizing antibodies or small molecules were observed or designed to lock the close conformation of the spike.^30,63^ However, our finding showed that the non-ACE2 competing antibody n3113 stabilized a 3-up conformation of spike and neutralized SARS-CoV-2 pseudoviruses, advancing the understanding of the neutralizing mechanism by antibodies against SARS-CoV-2.

The binding epitope of n3113 is partially covered by N-glycans at residues N165, thus considered as a ‘silent face’.^64^ The hindrance for B cell receptor access caused by glycans at N165 may rule out the generation of antibodies binding at such epitope through natural immunity. N3113 was selected through bio-panning using the SARS-CoV-2 S1 domain as target antigen in a phage display library. The fast and versatile platform can therefore replenish the antibody repertoire by overstriding the obstruction. It was suggested that antibodies that bind to RBM and compete with ACE2 elicited higher neutralizing potency.^65^ However, residues on RBM bear high evolutionary pressure and undergo high mutation during virus circulation. Furthermore, we found previously that the natural SARS-CoV-2 variants had a higher tendency to escape the binding of ACE2-competing antibodies than non-competing antibodies.^66^ The epitope of n3113 is away from the RBM and mutations are substantially less frequently found. N3113.1 maintains neutralizing potency to the recently spreading lineages worldwide (Fig. 4). With the less immunogenicity risk and the small size of n3113, by inhaling directly, it could reach the pulmonary organ, potentially penetrating the brain blood-barrier. The development of n3113 and its variants may provide a new clinical therapy to compensate the mAbs against SARS-CoV-2 and shed light on the amelioration of lineage escape.

## Supplementary information

Supplementary materials

## Data Availability

The atomic coordinate and structure factor amplitude for the RBD-n3113 complex have been deposited in the Protein Data Bank (PDB, http://www.rcsb.org/) under accession code 7VNB. The atomic models generated from cryo-EM studies of the n3113-S 2 P (UDD, state 1), n3113-S 2 P (UUD, state 2), and n3113.1-S 2 P (UUU) are deposited in the Protein Data Bank under accession codes 7VNC, 7VND, and 7VNE, respectively. The corresponding EM density maps have been deposited to the Electron Microscopy Data Bank under the accession codes EMD-32038 (UDD, state 1), EMD-32039 (UUD, state 2), and EMD-32040 (UUU). All reagents and relevant data are available from the corresponding authors upon reasonable request.
