# Supplementary material for: A non-ACE2 competing human single-domain antibody confers broad neutralization against SARS-CoV-2 and circulating variants
Source: Signal Transduct Target Ther. 2021 Nov 3;6:378. doi: 10.1038/s41392-021-00810-1 (PMC8564274; doi:10.1038/s41392-021-00810-1)
Supplement: Supplementary file 1 — Supplementary materials [file 41392_2021_810_MOESM1_ESM.docx]

**Supplementary Materials for**

**A non-ACE2 competing human single-domain antibody confers broad neutralization against SARS-CoV-2 and circulating variants**

Zhenlin Yang^1,2,3#*^, Yulu Wang^4#^, Yujia Jin^4#^, Yuanfei Zhu^4#^, Yanling Wu^2,4#^, Cheng Li^4^, Yu Kong^4^, Wenping Song^4^, Xiaolong Tian^4^, Wuqiang Zhan^5^, Ailing Huang^2,4^, Shanshan Zhou^4^, Shuai Xia^4^, Xiaoxu Tian^6^, Chao Peng^6^, Cuicui Chen^1,3^, Yibing Shi^4^, Gaowei Hu^4^, Shujuan Du^4^, Yuyan Wang^4^, Youhua Xie^4^, Shibo Jiang^4^, Lu Lu^4^, Lei Sun^5^, Yuanlin Song^1,3,7*^, Tianlei Ying^2,4,8*^

^1^Department of Pulmonary Medicine, Zhongshan Hospital, Fudan University, Shanghai 200032, China

^2^Shanghai Engineering Research Center for Synthetic Immunology, Shanghai 200032, China

^3^Shanghai Key Laboratory of Lung Inflammation and Injury, Shanghai 200032, China

^4^MOE/NHC Key Laboratory of Medical Molecular Virology, Shanghai Institute of Infectious Disease and Biosecurity, School of Basic Medical Sciences, Shanghai Medical College, Fudan University, Shanghai 200032, China

^5^The Fifth People’s Hospital of Shanghai, Fudan University and Shanghai Key Laboratory of Medical Epigenetics, Institutes of Biomedical Sciences, Fudan University, Shanghai 200032, China

^6^National Facility for Protein Science in Shanghai, Zhangjiang Lab, Shanghai Advanced Research Institute, Chinese Academy of Science, Shanghai 201210, China

^7^Department of Pulmonary Medicine, Shanghai Respiratory Research Institute, Shanghai 200032, China

^8^Lead Contact

^#^These authors contributed equally

^*^Correspondence to: tlying@fudan.edu.cn, yang_zhenlin@fudan.edu.cn, song.yuanlin@zs-hospital.sh.cn

**This PDF file includes:**

Materials and Methods

Figures. S1 to S10

Tables. S1 to S5

Materials and Methods

**Protein expression, purification and characterization**

The gene encoding for 319-531 of SARS-CoV-2 RBD was synthesized by Genwiz and was inserted into a modified pFastBac1 vector (Invitrogen), which contains HA signal signal peptide sequence before RBD and a PreScission protease site followed by a hexa-histidine tag at the C terminus. High-titer (>10^8^ viral particles per ml) recombinant baculovirus was generated by Bac-to-bac Baculovirus Expression System (Invitrogen). The baculovirus at an MOI (multiplicity of infection) of 5 was then used to infect *Spodoptera frugiperda* (*Sf*9) cells and the infected cells were cultured for 2 days at 27 ℃ to express RBD_319-531. The cell culture was centrifuged and the supernatant with secreted RBD_319-531 was filtered and incubated with Ni-NTA resin (GE healthcare) at 4 ℃ for 3 h. The resin was collected and washed in buffer containing 25 mM HEPES, pH7.5, 200 mM NaCl and 25 mM imidazole extensively to remove contaminants. The target protein was then eluted in buffer containing 25 mM HEPES, pH 7.5, 200 mM NaCl and 250 mM imidazole. The purified protein was diluted in 25 mM HEPES, pH 7.5 and 200 mM NaCl to remove imidazole and digested overnight using Precision Protease (custom-made) and PNGaseF (custom-made). Ni-NTA resin (GE healthcare) reverse binding was performed to remove the PreScission protease and the cleaved His-tag. RBD_319-531 was collected and analyzed by SDS-PAGE.

Recombinant RBD and RBD mutants that used for testing the binding to n3113 were expressed in Human Embryonic Kidney (HEK) 293 Freestyle cells (Invitrogen). Gene of RBD (319-541) was fused with the Fc fragment of human IgG1 and cloned into mammal expression vector pSeqtag containing an N-terminal murine Igκ chain leader sequence. Plasmid of the mutated RBD was constructed using the parental one as template and mutated through QuickMutation™ Site-Directed Mutagenesis Kit (Beyotime) following circular PCR. The plasmid was transfected into HEK293 cells and incubated at 37°C for 4 days. The supernatant with secreted protein was collected and purified by Protein G Resin (GenScript) according to the manufacture’s protocol.

SARS-CoV-2 S2P spike and variants were produced as described previously^1^. The gene for S2P spike encodes 1-1208 of WH01 ECD, with mutations of RRAR (682-285) to GSAS to remove the furin cleavage site, and 986-987 to proline to stabilize the protein. Plasmid of the mutated spike was constructed following the same procedure as mentioned above for RBD mutations. The gene was inserted into a modified pcDNA3.1 plasmid containing expression cassette with C-terminal precision protease site followed by twin strep tag and octa-histidine tag. The expression constructs were transiently transfected into HEK293 (cell density of 2.0 × 10^6^/mL) and incubated at 37°C for 4 days. The cells were centrifuged and the expressed spike protein was collected and purified following the same nickel affinity chromatography procedure as used for the RBD protein purification.

The DNA sequences of single domain antibodies was cloned into pComb3x vector with N-terminal OmpA signal peptide (MKKTAIAIAVALAGFATVAQA) and C-terminal hexahistidine and Flag tag and was expressed in *E. coli* HB2151 cells. SB medium containing 100 µg/µl ampicillin was cultured to *A*_600nm_ ~ 0.6 and 1 mM IPTG was added to induce expression. After 14 hours expression at 30 ℃, the cells were collected, resuspended in PBS buffer and disrupted by ultrasonication. The supernatant was collected after centrifugation at 17,000 g for 30 minutes and incubated with Ni-NTA (GE healthcare). The resin was washed in PBS buffer containing 25 mM imidazole and antibody was eluted by PBS buffer supplemented with 250 mM imidazole.

***In vitro* affinity maturation of n3113**

A large phage display library, with an estimated diversity of 6 × 10^9^, was constructed by introducing randomly mutation into the full-length gene of n3113 though four rounds of error-prone PCR (Mutazyme, Agilent Technologies). The mutagenized PCR product was amplified and cloned into the pComb3x phagemid as described previously^2,3^. The library was panned using biotin labeled SARS-CoV-2 RBD (residues 331 to 527) conjugated to magnetic beads (Invitrogen). Amplified libraries with 10^12^ phage-displayed n3113 mutants were used in the following bio-panning. Four rounds of panning were performed as described above^2,3^. After panning, the enriched output clones that bound to SARS-COV-2 RBD-Fc protein were randomly picked from the fifth selection round bio-panning using monoclonal phage ELISA. The selected clones were sequenced, and 4 dominant clones were identified.

**Protection against SARS-CoV-2 in hACE2 mice**

A total of 10 hACE2 transgenic mice (GemPharmatech) were randomly split into two groups as therapeutic or prophylactic (one mouse died before the experiment) group and negative control, respectively. All hACE2 mice were inoculated intranasally with 5 × 10^4^ PFU SARS-CoV-2 virus, which was performed under anesthesia to minimize animal suffering. The mice were intraperitoneally (2 hours after infection) treated with 40 mg/kg n3113.1-Fc for treatment or the same volume of PBS for negative control. Animals were sacrificed at 5 dpi and lung tissues were harvested for viral load analysis.

Measurement of viral burden in lung tissue was performed by qRT-PCR. The lung tissue was first collected and homogenized by electric homogenizer in Trizol. After centrifugation, the total RNA was extracted from the supernatant by chloroform and isopropanol and was reversely transcribed into cDNA. Each mouse was quantitated with three replicates by qRT-PCR kit (TianGen) using primers that target a conserved region in necleocapsid (N) gene of SARS-CoV-2. qRT-PCR of serial diluted N gene with known copies was performed to generate quantitative standard curve.

**Crystallization and data collection**

The purified recombinant RBD protein and n3113 antibody were mixed at molar ratio of 1:1.5 for 1 hour at 4 degree and the complex was subjected into a Superdex 200 10/300 GL column with 20 mM HEPES, pH 7.5 and 100 mM NaCl as flowing phase. The peak of complex protein was collected and concentrated to 10 - 20 mg/ml for crystallization.

Crystals of RBD-n3113 complex were obtained with the sitting drop method by mixing 0.2 ul of reservoir solution (4.3M sodium chloride and 0.1M HEPES, pH 7.5) (Hampton Research) and 0.2 μl protein (10 mg/ml). Crystals were harvested and flash-frozen in liquid nitrigon using 20% glycerol as cryo-protectant. The X-ray diffraction data was collected at 100 K in beamline BL17U1 and BL19U1 in Shanghai Synchrotron Radiation Facility, Chinese Academy of Sciences^4,5^.

**Determination and refinement of crystal structure**

Diffraction images of RBD-n3113 were indexed and processed by HKL2000^6^. The structure of RBD-n3113 was solved by molecular replacement implemented in the Phaser program in CCP4 crystallography package^7^ using RBD (RBD isolation from PDB entry 6LZG) and a camelid-derived single-domain antibody (PDB ID code 3K1K) as search models. Refmac^8^ and Phenix^9^ were used to refine the structure. The model was further adjusted by COOT^10^. The crystallographic parameters of RBD-n3113 complex (PDB ID 7VNB, 2.27Å) are shown in supplementary information, Table 2. The related structural analysis figures were drawn by PyMOL^11^.

**Cryo-EM sample preparation and data acquisition**

SARS-CoV-2 S2P spike was incubated with single domain antibody n3113 or n3113.1 at molar ration of 1:1.2 for 30 minutes on ice. 3 μl of 1 mg/ml complex sample was applied to glow-discharged Quantifoil holey carbon grids (R1.2/1.3, 300 mesh, gold). Grids were blotted for 3.5s under 100% relative humidity at 20 °C, and plunged into liquid ethane using a Mark IV Vitrobot (FEI). Grids were imaged on a Titan Krios electron microscope (FEI) operated at 300 kV with a K2 Summit direct electron detector (Gatan). Datasets were collected using the automated data collection program SerialEM in counting mode with a pixel size of 1.01 Å. Each micrograph was acquired at an exposure time of 8s and dose-fractionated to 40 frames under a dose rate of 8 e-/pixel/s. The defocus range was set from -1.2 μm to -2.5 μm.

**Image processing**

The motion correction was performed using the MotionCorr2, and the contrast transfer function parameters of the micrographs were determined using the GCTF program. All other following steps of image processing were performed using RELION3.0. For n3113-S protein, 502,388 particles were automatically picked from 1,906 micrographs for 2D classification. Good particles were selected for two rounds of 3D classifications. Three major classes, i.e, two RBDs in open state (UUD), one RBD in open state (UDD) and all RBDs in close state (DDD), were selected for 3D refinement to final resolution of 3.55 Å, 3.7 Å and 3.6 Å, respectively. The data processing procedures of n3113.1-S were similar to that of n3113-S. The data processing details are summarized in Supplymentary information, Fig.4 and 5.

**Enzyme-linked immunosorbent assay (ELISA)**

Purified protein (RBD-Fc) at 100 ng per well in PBS was coated in costar half-area high binding assay plate (Corning #3690) at 4 ºC overnight, and then blocked with PBS containing 5% BSA for 1 h at 37 ºC. The plate was washed three times with PBST (PBS containing 0.05% Tween 20), and 50 μl of three-fold serially diluted single antibody solutions in PBS starting at 2 μM was added. The antigen-antibody mixture was incubated for 1.5 h at 37 ºC and washed for three times with PBST. 50 μl of HRP-conjugated anti-Flag secondary antibody (Sigma-Aldrich) was added for another 45 min at 37 ºC. The enzyme activity was measured by recording the absorbance at 405 nm using a Microplate Spectrophotometer (Biotek, Hercules, CA, USA) after incubating with ABTS (Invitrogen) for 15 minutes. The data was plotted using Graphpad Prism (GraphPad, San Diego, CA). The standard deviation of triplicates was added as error bars.

**Biolayer interferometry (BLI) assay**

The binding kinetics of n3113.1 and n3113.1-Fc to spike and spike variants mutant was measured by BLI on an Octet-Red 96 (Pall ForteBio, Sartorius, Germany). Briefly, the recombinant spike at 10-30 μg/ml in 10 mM sodium acetate (pH 5.0) was immobilized onto activated Amine Reactive Second-Generation (AR2G) biosensors until saturation. Loaded biosensors were quenched in 10 mM ethanol amine (pH 8.5) and were dipped into wells containing threefold serial dilutions of single-domain antibodies in kinetics buffer (PBS buffer supplemented with 0.02% Tween 20). Baseline was established in kinetics buffer. Spike: antibody complexes were then allowed to dissociate in kinetics buffer. The procedures of the experiment were as follows at 37 ºC: (1) equilibration for 60 s; (2) activation of AR2G by 1-ethyl-3-(3-dimethylaminopropyl) carbodiimide hydrochloride/N-hydroxysucci-nimide (300 s); (3) immobilization of S protein onto sensors (300 s); (4) quenching with ethanolamine (300 s); (5) baseline in kinetics buffer (300 s); (6) association of antibodies for measurement of *k*_on_ (300 s); and (7) dissociation of antibodies for measurement of *k*_off_ (300 s). The curves were fitted by a 1:1 or 1:2 binding model using the Data Analysis software 10.0. Mean *k*_on_, *k*_off_, and K_D_ values were determined by averaging binding curves within a dilution series having R^2^ values of greater than 95% confidence level. Graph was made using Graphpad Prism (GraphPad, San Diego, CA).

Binding of n3113 to its escape RBD mutant L452R was performed by BLI using streptavidin-coated biosensors. RBD was pre-biotinylated using EZ-Link™ NHS-Biotin Reagents (Thermo) according to the manufacturer’s protocol. Biosensors were immobilized with biotinylated RBD mutants or wild type RBD and then incubated with 10 μg/ml antibodies. The procedures of the experiment were as follows at 37 ºC: (1) equilibration for 60 s; (2) immobilization of biotinylated 10 μg/mL RBD-Fc mutants or wildtype RBD-Fc diluted with kinetics buffer onto sensors for 120 s; (3) baseline in kinetics buffer for 300s; (4) antibodies association for 300s or 600s; (5) antibodies dissociation for another 300s or 600s. The curves were connected using Graphpad Prism (GraphPad, San Diego, CA).

**Binding Competition Assays**

Sensor tips loaded with SARS-CoV-2 RBD were immersed into wells containing the antibodies at a concentration (1 µM n3113.1 or 100 nM n3113.1-Fc, CB6 and S309) necessary to reach binding saturation after 300 s. Next, biosensors were dipped into wells containing the first antibody in the presence of or without 200 nM ACE2 (Novoprotein Scientific Inc., Shanghai), and binding was measured after 600 s of association. The signal obtained for binding of ACE2 in the presence of the first antibody was expressed as a percentage of the uncompeted binding of ACE2 that was derived independently. A residual binding of <30%, 30%-70% and >70% are considered as non-competition, intermediate competition and strongly competition, respectively.

**Pseudovirus neutralization assay**

To determine the neutralization activity of n3113.1-Fc, the pseudotyped virus neutralization assay was performed following a well-established pseudovirus neutralization assay^12^. Briefly, pseudoviruses bearing SARS-CoV-2 spike glycoprotein derived from wild type strain, or variants of B.1.1.7, B.1.351, P.1, P.1.617.2 and C.37 that declared by the Centers for Disease Control and Prevention (CDC), and a defective HIV-1 genome that encompasses luciferase reporter were produced in HEK293 T cells (ATCC, Manassas, VA, USA), and supernatants containing pseudoviruses were collected 48 hours post-transfection. Threefold serial dilutions of antibodies were incubated with pseudoviruses at 37°C for 1 hour, and then the mixtures were added into ACE2 expressed Huh-7 cells (10^4^ per well in 96-well plates). The culture medium was refreshed 12 hours post-infection and followed by an additional 48 hours incubation. Huh-7 cells were subsequently lysed with 50 μL lysis reagent (Promega), and 30 μL of the lysates were transferred into 96-well Costar flat-bottom luminometer plates (Corning Costar) for the detection of relative light units using the Firefly Luciferase Assay Kit (Promega) on Ultra 384 luminometer (Tecan). A nonlinear regression analysis was performed on the resulting curves using Graphpad Prism (GraphPad, San Diego, CA) to calculate half-maximal inhibitory concentration (IC50) values. All cell lines were cultured in Dulbecco’s modified Eagle’s medium (DMEM) with 10% fetal bovine serum (FBS).

**Flow cytometry–based S protein binding assay**

His-tagged S protein binding to hACE2 on the cell surface was measured by flow cytometry. 1 × 10^5^ HEK-293T cells engineered to express hACE2 or Huh7 cells endogenously expressing hACE2 were detached and washed with PBS. Cells were resuspended in 100 μl PBS and Zombie Viability Kit (catalog no. 423113, Biolegend) was added to the cells, followed by incubation at room temperature for 15 minutes. After thorough washes, 10 μg/ml his-tagged S trimer, or S trimer pre-incubated with antibodies at a molar ratio of 1:10 were added to the cells in fluorescence-activated cell sorting (FACS) buffer (PBS containing 20 g/L bovine serum) and incubated at 4 ℃ for 1h. Cells were washed with FACS buffer and dyed with PE-conjugated anti-his Ab (catalog no. 362603, Biolegend) for an additional 30 mins at 4 ℃. After washing, cells were fixed in PBS and the antibodies bound to the stained cells were analyzed in a flow cytometer (AttuneTM, Thermo) using a FlowJo software (BD bioscience).

**Cell-cell fusion assay**

Cell-cell fusion assay was performed following the procedure described previously^13,14^. 293T cells at density of 3 × 10^5^ per well were cultured in DMEM for 12 hours and transfected with pAAV-SARS-CoV-2 S-EGFP or empty plasmid pAAV-IRES-EGFP. The cells were harvested 48 hours after transfection and used as effector cells. 100 μL effector cells (1 × 10^5^/mL) were mixed with or without antibodies for 5 minutes, and the mixtures were transferred to the Huh-7 cells (5,000 per well) that express ACE2. Medium was changed after 12 h and incubation was continued for 48 h at 37 ℃. After incubation, cells were harvested and fixed with 4% paraformaldehyde (PFA) at room temperature for 15 minutes. The extra PFA was washed by PBS for two times. Nuclei was stained with 4,6-diamidino-2-phenylindole (DAPI). The fluorescence images were recorded by an inverted fluorescence Microscope (AMG, USA).

**HDX MS (hydrogen-deuterium exchange mass spectrometry) assay**

**Sample Preparation**

Protein samples were processed automatically by a LEAP Technologies Hydrogen Deuterium Exchange PAL system (Carrboro, NC). Specifically, 3.0 μL of each sample (spike, 2 μg/μL; n3113.1-Fc, 2 μg/μL) was automatically dispensed into a vial and diluted 9-fold with 20m M HEPES, 150 mM NaCl in 99.8% D_2_O (pH read 7.4) to start the deuterium exchange reaction. HDX measurements were taken at 0 s, 30 s, 100 s, 300 s, 1000 s, 3000 s and 10000 s at 4 °C. After each time point, an aliquot of sample was transferred to a vial in a 0.5 °C chamber and quenched by addition of an equal volume of quench buffer (200 mM Citric acid, 4 M Gu-HCl, 500 mM TCEP in H_2_O, pH read 2.3) for 0.5 min prior to online digestion. The complete HDX-MS procedure was repeated three times for each sample and each time point.

**Online Digestion and LC-MS Analysis**

Each quenched sample was immediately injected into a Protease type XIII pepsin column (NovaBioAssays LLC, Woburn, MA) for 4 min at a flow rate of 50 μL/min with 0.1% formic acid in water then delivered by the loading pump on a Thermo Dionex Ultimate 3000 NCS- 3500RS system (Sunnyvale, CA). The digested peptides were trapped and desalted using a 2.1 × 5 mm Acclaim PepMap 300 C18 μ-precolumn (300 Å, 5 μm). The precolumn was connected to a 1.0 × 50 mm Thermo Hypersil Gold column C18 (175 Å, 1.9 μm). Peptides were eluted and separated by a linear gradient of Buffer B (0.1% formic acid in 80% acetonitrile) at a flow rate of 45 μL/min using the nanopump of the NCS-3500RS system. Specifically, the gradient was 4-10% over 3 min, 10-30% over 8 min, 30-90% over 1 min followed by isocratic flow with 90% Buffer B for 1 min. The online digestion, trapping, desalting process was performed at 4 °C and separation process was performed at 0.5 °C in the temperature-controlled compartment of the HDX PAL system. Data was acquired using a Thermo LTQ Orbitrap-Elite mass spectrometer (San Jose, CA) with a Thermo H-ESI II probe. For peptide identification, mass spectra were acquired in a data-dependent scan using FTMS mode in MS1 (one microscan, 100 ms max injection time, 60 k resolution at 400 m/z) at the m/z range of 300-1500 followed by ten CID MS2 scans in the ion trap with a ± 2.0 m/z isolation width. Once the peptides were identified, the deuterium uptake in HDX experiments was conducted using FTMS mode in MS1.

**Peptide Assignment**

The spectra generated were searched in PEAKS Studio X against an homemade database including target protein with a precursor mass tolerance of ≤20 ppm and MS/MS fragment ≤ 0.02 Da. Retention time and sequence information for each peptide were exported to Excel for HDX data processing.

**HDX Data Analysis**

HDX data analysis was carried out using HDExaminer 2.0 (Sierra Analytics Inc., Modesto, CA). The number of D taken up (D-uptake) by each peptide at each exchange time was calculated by the software algorithm for matching the best theoretical isotope distribution pattern to the observed isotope distribution pattern. D-uptake was plotted as a function of exchange time. Triplicate runs were compared using Student’s t-test at the 95% confidence level to confirm the consistency of the analytical results obtained. D-uptake was converted to %D for each peptide based on the theoretical number of D; %D was used to generate heat maps, butterfly comparisons, and difference plots. In addition, H/D-ex analysis was also carried out on non-deuterated and fully deuterated samples to correct back-exchange^15^.

**Statistical analyses**

Statistical analyses were performed using GraphPad Prism version 8 (GraphPad Software Inc, USA). Individual or multiple group comparisons were performed by one-tailed unpaired Student’s t-test. P values < 0.05 were considered statistically significant, and different levels were denoted as *P< 0.05, **P<0.01, and ***P< 0.001, respectively.


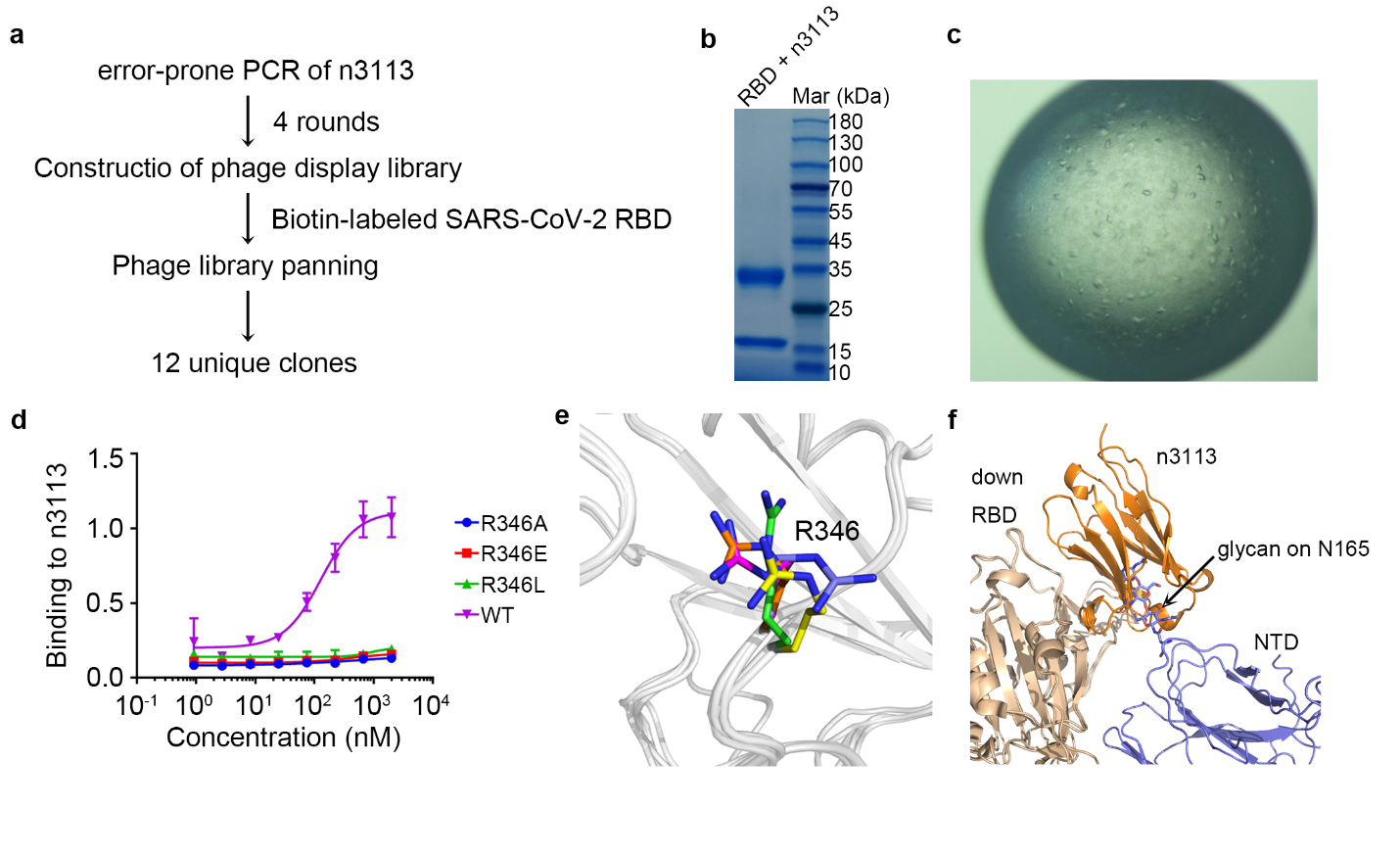


Figure. S1.

**Scheme of n3113 maturation, co-crystallization sample for n3113-RBD and structural features of n3113-RBD. a.** Scheme of n3113 maturation by screening of phage-display library. **b**-**c**. SDS-PAGE (**b**) and crystals (**c**) of n3113-RBD complex. **d**. Binding of n3113 to RBD-WT, RBD-R346A, RBD-R346E and RBD-R346L was tested by ELISA. All data are shown as mean ± SD from three independent experiments. **e**. Side chains of R346 in RBD-n3113, RBD-ACE2 (PDB entry 6LZG), RBD-ACE2 (PDB entry 6M0J), RBD-2F6 (PDB entry 7BWJ) and RBD-B38 (PDB entry 7BZ5) are shown as sticks colored in blue, magenta, orange, yellow and green, respectively. **f**. Superimpose of crystal structure of RBD-n3113 into the close-state RBD in cryo-EM structure of spike-n3113. The close RBD is colored in brown. N3113 is shown as orange cartoon. The adjacent NTD is represented as blue cartoon with the side chain and glycan of N165 shown as sticks.


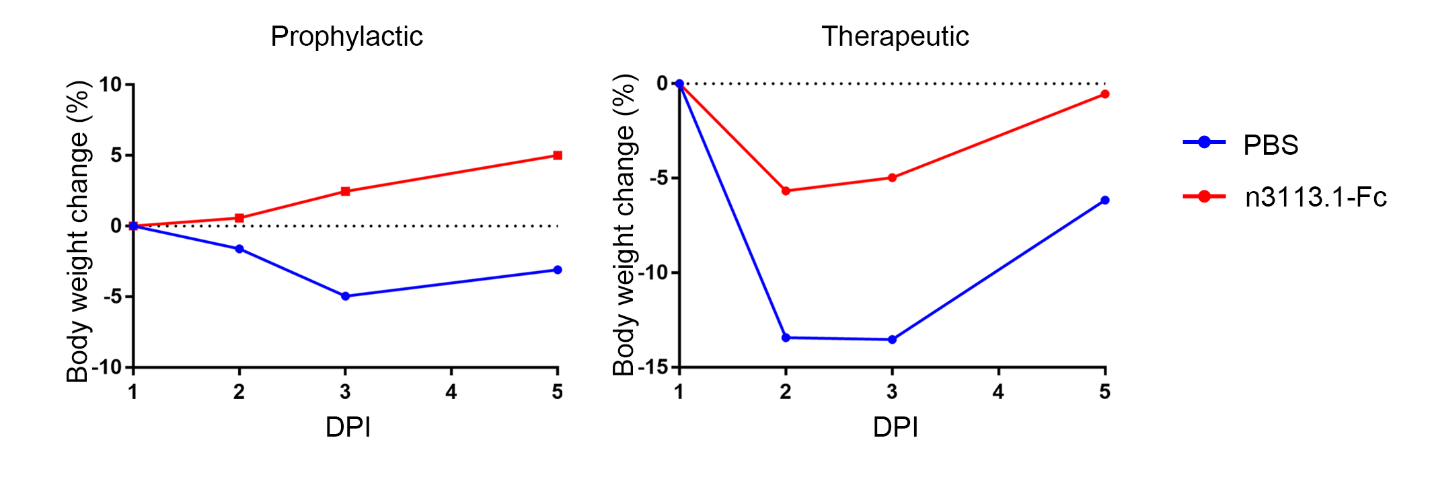


Figure. S2.

**Body-weight change (%) of the hACE2 transgenic mice recorded over 5 days.** Each group contains 5 mice except for the group that received prophylactic treatment of n3113.1-Fc, which contains 4 mice. Mean value of weight is presented.


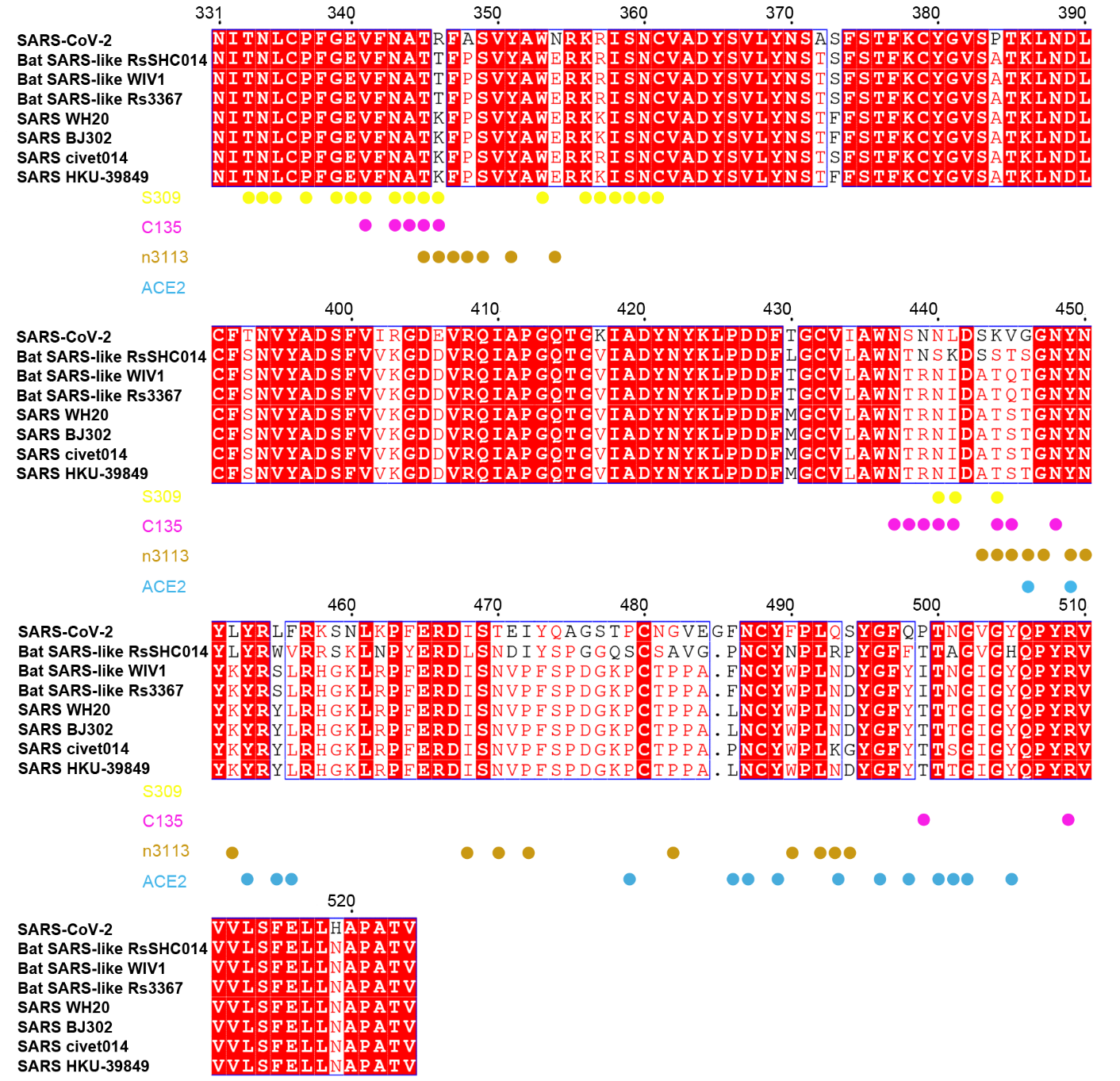


Figure. S3.

**Sequence alignment of RBDs in coronaviruses and epitopes of side-surface-binding neutralizing antibodies and ACE2 on SARS-CoV-2 RBD.** The colored dots indicate the epitopes of C135 (magenta), S309 (yellow), n3113 (orange) and ACE2 (cyan) on SARS-CoV-2 RBD.


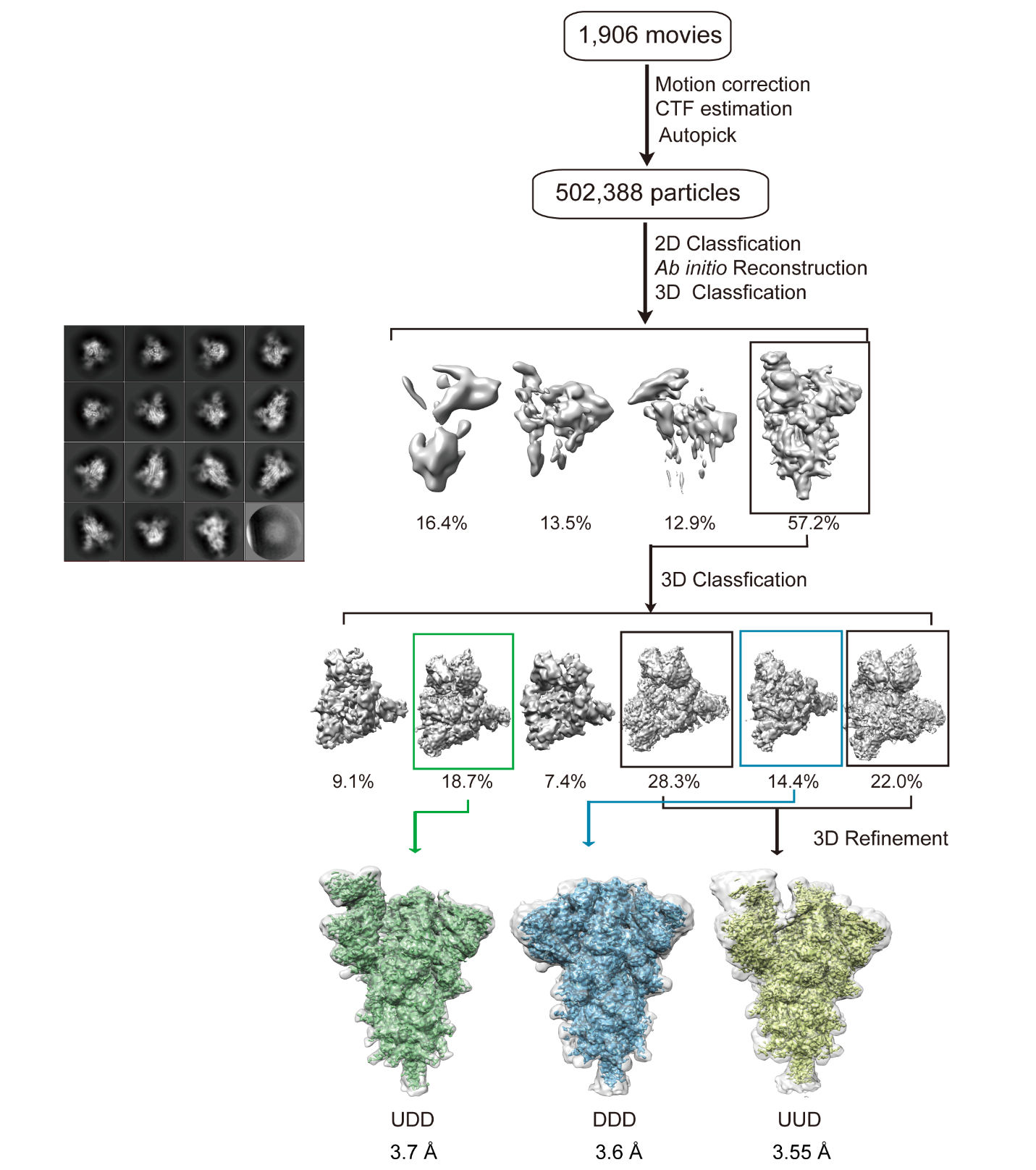


Figure. S4.

**Flowchart of cryo-EM data processing of SARS-CoV-2 S trimer in complex with n3113.**

**
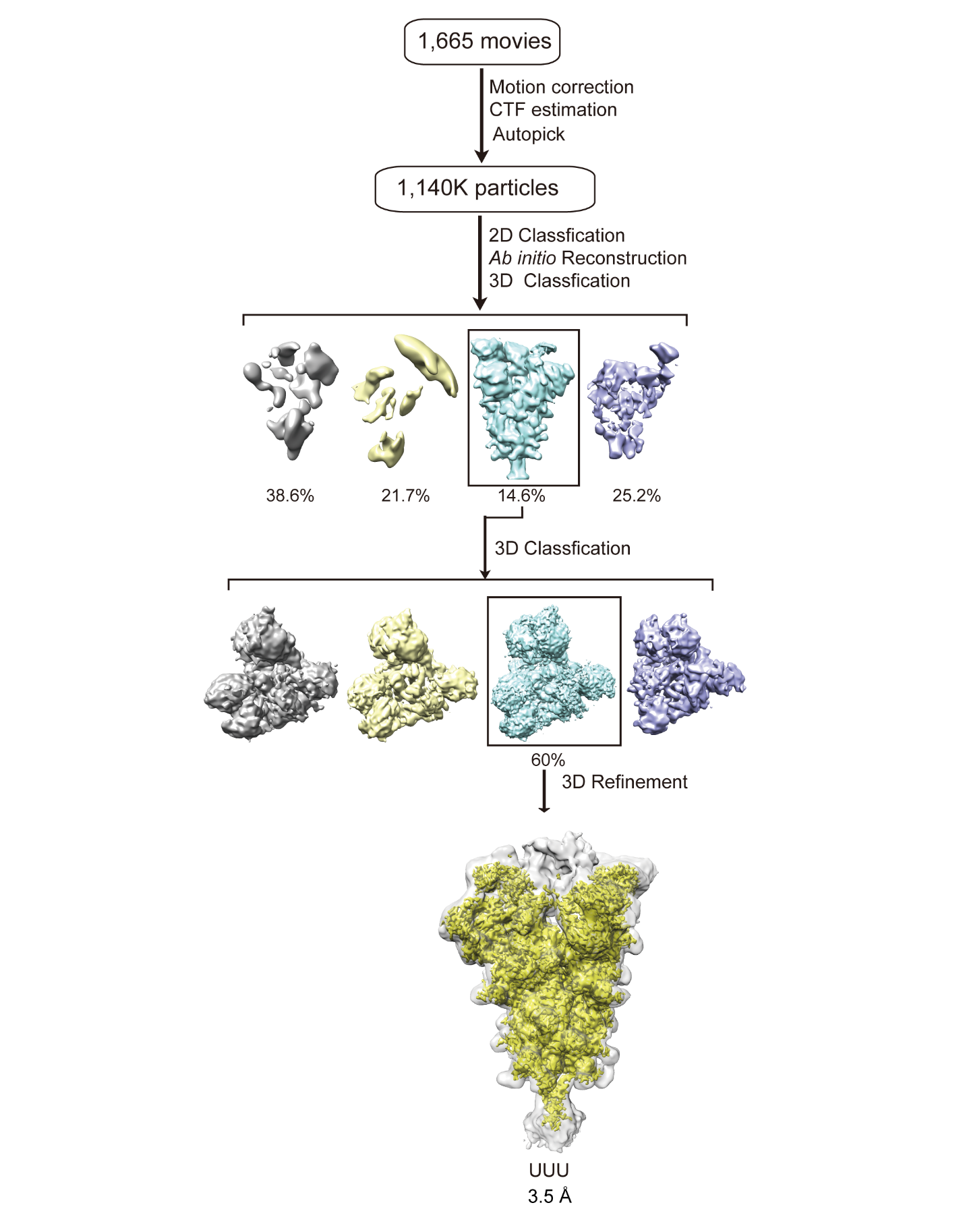
**

Figure. S5.

**Flowchart of cryo-EM data processing of SARS-CoV-2 S trimer in complex with n3113.1.**


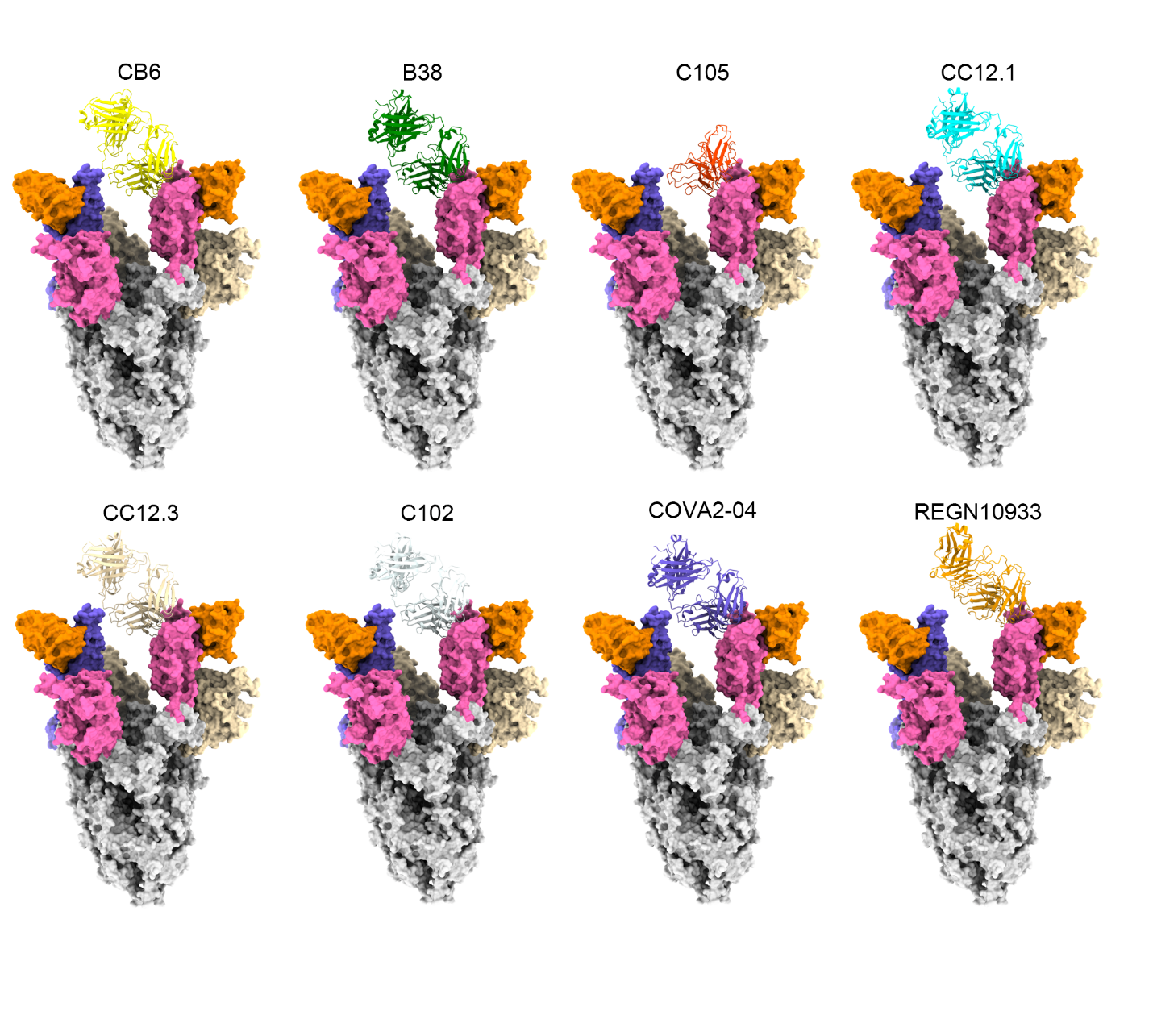


Figure. S6.

**Superimpose of SARS-CoV-2 neutralizing antibodies with the UUD-state S-n3113 structure.** Fab of CB6 (PDB entry 7C01), B38 (PDB entry 7BZ5), C105 (PDB entry 6XCN), CC12.1 (PDB entry 6XC2), CC12.3 (PDB entry 6XC4), C102 (PDB entry 7K8M), COVA2-04 (PDB entry 7JMO), REGN10933 (PDB entry 6XDG) and Fab fragment of C105 (PDB entry 6XCN) are shown as cartoon colored in yellow, green, cyan, brown, grey, blue, orange and red, respectively. Different components in S-n3113 are represented as surface colored as indicated in Fig. 2**f**.


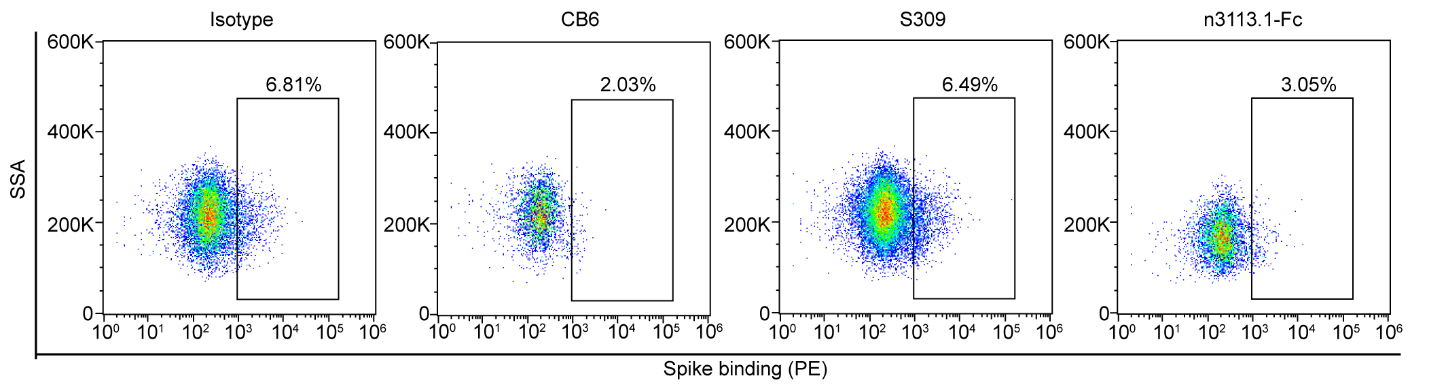


Figure S7.

**Analysis of S protein attachment to Huh7 cells.** Huh7 cells were stained with 10 µg/mL his-tagged SARS-CoV-2 spike proteins pre-incubated with isotype IgG, CB6 or n3113.1-Fc. The percentage of binding was measured by anti-histag PE and analyzed by FACS. The experiments were performed twice with similar results and a representative figure was shown.


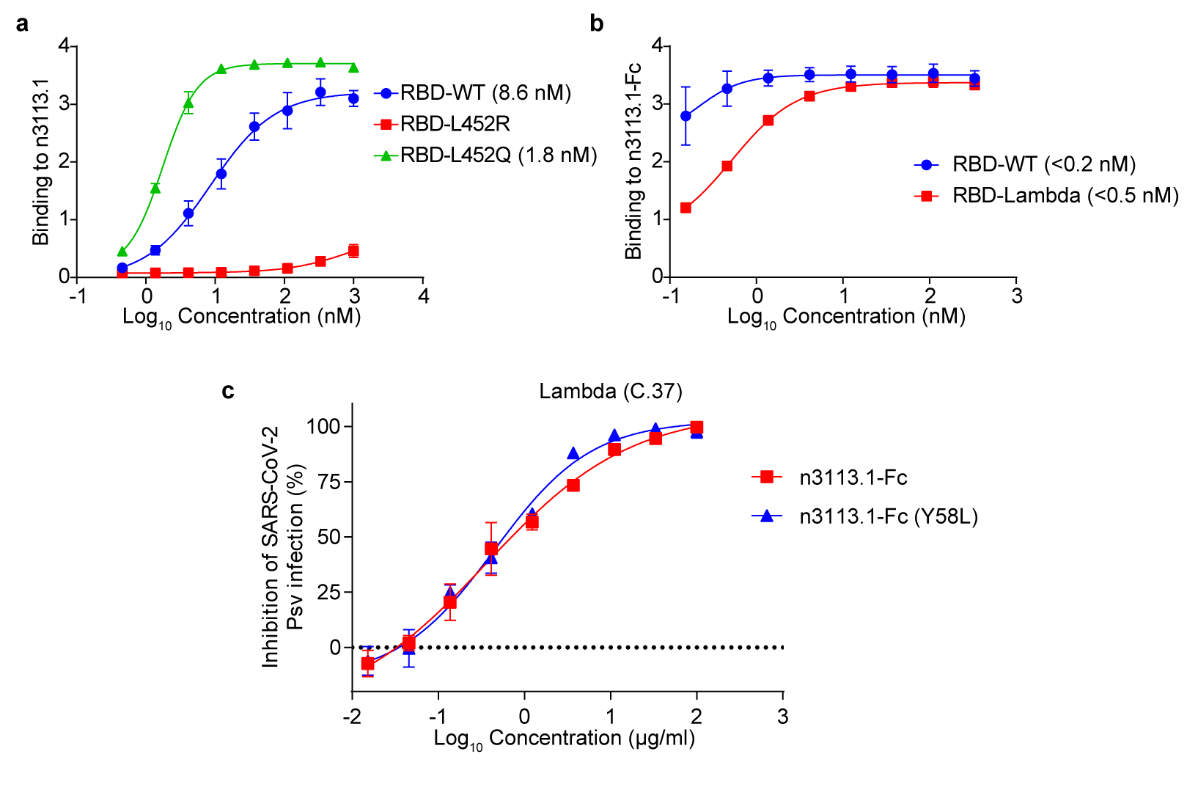


Figure S8.

**Binding and neutralization of variants of SARS-CoV-2. a**. Binding of n3113.1 to RBD-WT, RBD-L452R and RBD-L452Q was tested by ELISA. All data are shown as mean ± SD from four independent experiments. **b.** Binding of n3113.1-Fc to RBD-WT and RBD-L452Q/F490S (Lambda) was tested by ELISA. The experiments were performed in duplicate and mean ± SD data is represented. **c.** Neutralization profiles for n3113.1-Fc and n3113.1-Fc (Y58L) against pseudoviruses loaded with S of Lambda variant. The mean ± SD from three independent experiments is shown.


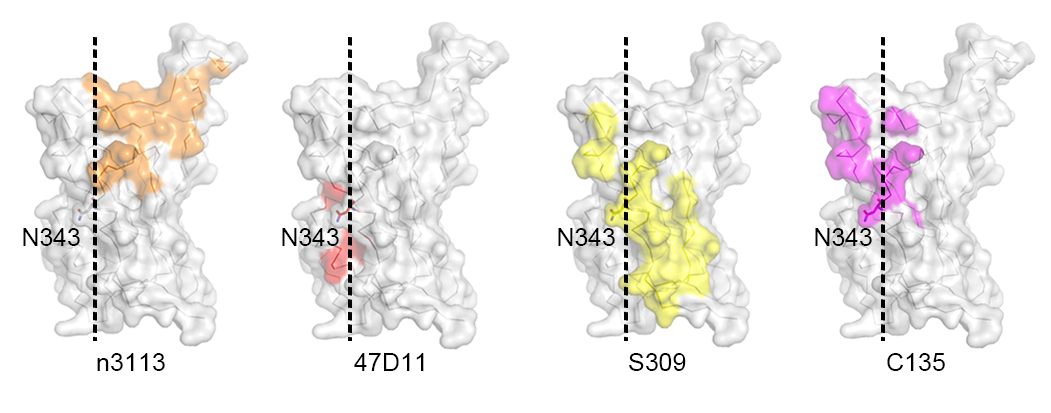


Figure S9.

**Epitopes of antibodies on ACE2.** Epitopes of n3113, 47D11 (PDB entry 7AKD and ref. 39), S309 (PDB entry 6WS6) and C135 (PDB entry 7K8Z) on RBD are highlighted in orange, red, yellow and magenta, respectively. The SARS-CoV-2 RBD is shown as grey cartoon and ribbon. Side chains of N343 are represented in sticks.


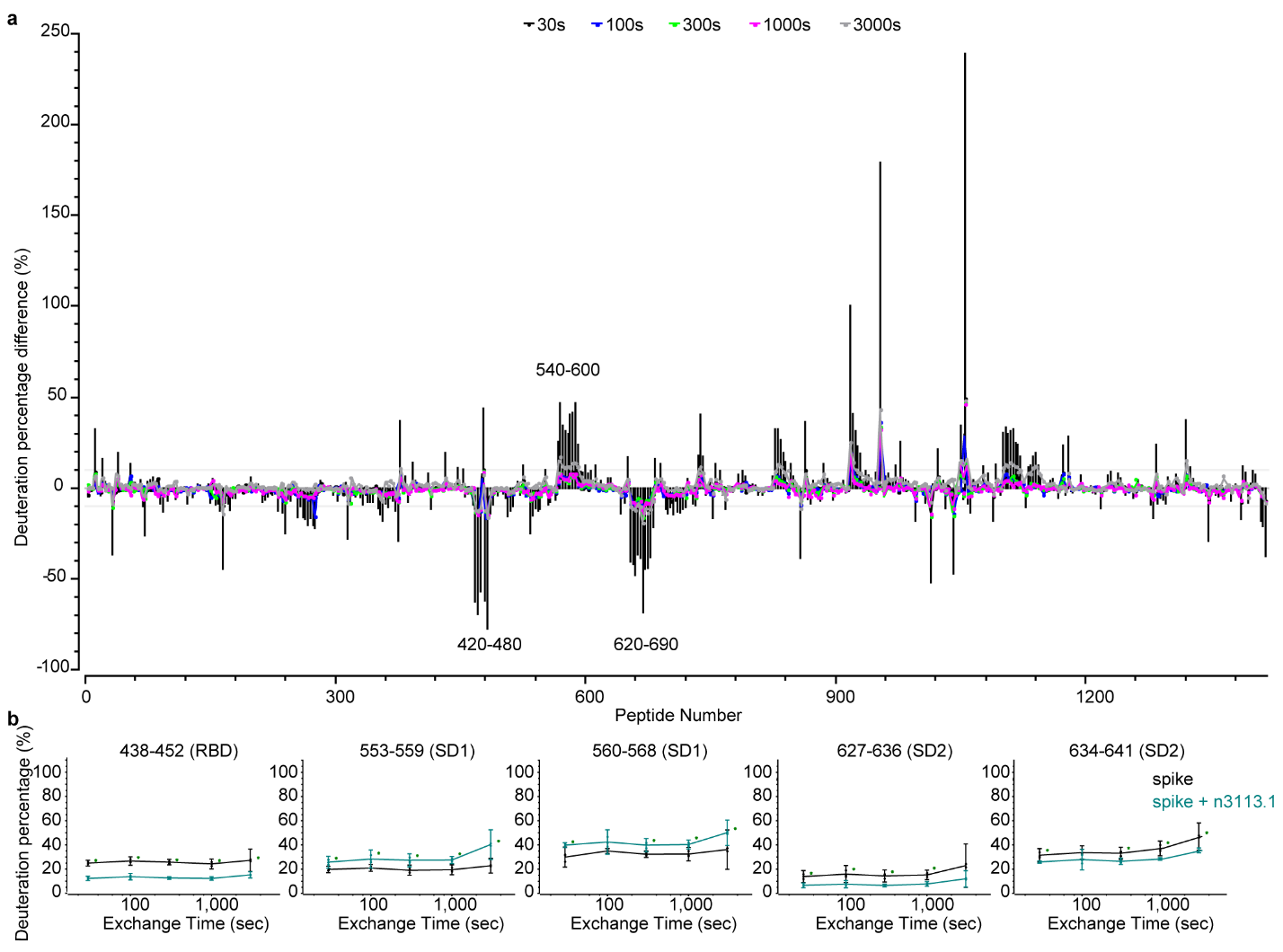


Figure S10.

**Differential HDX kinetics plots of the spike and spike-n3113.1 complex. a**. Deuterium uptake was monitored at 30s, 100s, 300s, 1000s and 3000s are presented as black, blue, green, magenta and grey lines, respectively. Accumulative deuterium uptake differences across the four time points were calculated and displayed as black bars. **b**. HDX kinetics of five peptides in spike. The uptake plot shows deuteration over time. The x‐axis shows deuteration time. The y‐axis shows the peptide’s deuteration percentage by default. Apo spike is colored in black and n3113.1 bound spike is colored in cyan. The asterisk represents a statistically significant difference.

Table S1.

**RBD binding affinity and SARS-CoV-2 S pseudovirus neutralization potency of n3113 and its variants. Related to Fig. 1a-b.**

|  | n3113 | n3113.1 | n3113.2 | n3113.3 | n3113.4 | n3113.1-Fc |
| --- | --- | --- | --- | --- | --- | --- |
| Binding to RBD-Fc (nM) | 132.2 | 4.4 | 9.6 | 24.4 | 12.4 | N.D. |
| Neutralization of SARS-CoV-2 (IC_50_) (μg/ml) | 11.0 | 5.8 | 3.4 | 2.8 | 6.0 | 0.06 |

N.D., not determined. All data are shown as means from three independent experiments.

Table S2.

**Data collection and refinement statistics of RBD-n3113**

| **Data collection** |  |
| --- | --- |
| Wave length (Å) | 0.9791 |
| Space group | *R3:H* |
| Cell dimensions |  |
| *a*, *b*, *c* (Å) | 146.2, 146.2, 93.1 |
| α, β, γ (º) | 90.0, 90.0, 120.0 |
| Resolution (Å) | 73.11-2.27 (2.33-2.27)^a^ |
| *R*_merge_ (%) | 18.5 (125.1) |
| *R_pim_* (%) | 6.1 (41.3) |
| *I/σ* (*I*) | 11.1 (2.2) |
| Completeness (%) | 99.9 (99.8) |
| Multiplicity | 10.4 (10.1) |
|  |  |
| **Refinement** |  |
| Resolution (Å) | 42.56-2.27 |
| No. reflections | 34,237 (2,535) |
| *R_work_ / R_free_* (%) | 17. 7/20.3 |
| Number of non-hydrogen atoms |  |
| Macromolecules | 2,600 |
| Ligands | 14 |
| Solvent | 245 |
| Average B-factor (Å^2^) | 40.4 |
| Macromolecules | 39.8 |
| Ligands | 52.7 |
| Solvent | 45.8 |
| R.m.s. deviations |  |
| Bond lengths (Å) | 0.008 |
| Bond angles (º) | 0.954 |
| Ramachandran^b^ |  |
| Favored (%) | 96.3 |
| Allowed (%) | 3.7 |
| Outliers (%) | 0.0 |

a, Values in the parentheses refer to the highest resolution shell.

b, As calculated by the Molprobity validation server.

Table S3.

**Residues of n3113 interacting with RBD^a^**

| n3113 | | RBD |
| --- | --- | --- |
| Location | Residues |  |
| CDR1 | Y32 | A352, I468 |
| FR2 | A44 | K444 |
|  | L45 | N450 (H^b^) |
|  | W47 | N450 (H), L452 |
| CDR2 | Y52 | Y351 (H), T470, F490, L492 |
|  | S54 | T470 (H) |
|  | R56 | T470 (H), G482 (H), I472, F490 |
| FR3 | Y58 | L452, F490, Q493, S494 (H) |
|  | N60 | Y449 |
|  | P61 | Y449, S494 |
|  | S62 | Y449 |
| CDR3 | W99 | R346 (H), N450 |
|  | A100 | S349 (H), Y351, A352 |
|  | S101 | A348, N354 (H) |
|  | G102 | R346, F347, A348 |
|  | S103 | T345 (H), R346 (H) |
|  | T104 | A344 |
|  | D106 | R346 (S^c^) |

a, d < 4 Å for hydrophobic interaction; d < 3.5 Å for polar interaction.

b, S indicates for salt bridge.

c, H indicates for hydrogen bond.

Table S4.

**Cryo-EM data collection and refinement statistics.**

|  | n3113-S (UUD) | n3113-S (UDD) | n3113.1-S (UUU) |
| --- | --- | --- | --- |
| **Data collection and processing** |  |  |  |
| Magnification | 49,310  300  64  -1.5 to -2.5  1.014  *C1* | | |
| Voltage (kV) |  |  |  |
| Electron exposure (e–/Å^2^) |  |  |  |
| Defocus range (μm) |  |  |  |
| Pixel size (Å) |  |  |  |
| Symmetry imposed |  |  |  |
| Initial particle images (no.) | 502,388 | 502,388 | 1,149,207 |
| Final particle images (no.) | 55,804 | 36,627 | 50,070 |
| Map resolution (Å)  FSC threshold | 3.55 | 3.7 | 3.5 |
|  |  |  |  |
| **Refinement** |  |  |  |
| Initial model used (PDB code) | *7ad1* | *7ad1* | *7ad1* |
| Map sharpening *B* factor (Å^2^) | -95.7 | -107.7 | -99.8 |
| Model composition  Non-hydrogen atoms  Protein residues  Ligands | 28,062  3,442  81 | 27,294  3,326  90 | 29,085  3,561  90 |
| *B* factors (Å^2^)  Protein  Ligand | 39.1  103.3 | 25.8  59.1 | 17.3  41.0 |
| R.m.s. deviations  Bond lengths (Å)  Bond angles (°) | 0.006  0.805 | 0.007  0.770 | 0.006  0.751 |
| Validation  MolProbity score  Clashscore  Poor rotamers (%) | 2.28  15.13  0.50 | 2.35  17.29  0.52 | 2.38  19.92  0.52 |
| Ramachandran plot  Favored (%)  Allowed (%)  Disallowed (%) | 88.1  11.95  0 | 87.64  12.27  0.09 | 88.43  11.54  0.03 |

Table S5.

**Information about spike variants and the apparent binding kinetic parameters between spike variants and n3113.1-Fc, related to Fig. 4b.**

|  | Mutations | K_D_ (M) | *k*_on_ (Ms^-1^) | *k*_off_ (s^-1^) | R^2^ value |
| --- | --- | --- | --- | --- | --- |
| S-D614G | D614G | 5.34E-09 | 1.17E+05 | 6.25E-04 | 0.99 |
| S-Alpha | 69-70del, 145Y, N501Y, A570D, ,D614G, P681H, T716I, S982A, D1118H | 4.82E-09 | 1.54E+05 | 7.39E-04 | 0.99 |
| S-Beta | L18F, D80A, D215G, 242-244del, R246I, K417N, E484K, N501Y, D614G, A701V | 1.01E-08 | 3.26E+05 | 3.28E-03 | 0.97 |
| S-Gamma | L18F, T20N, P26S, D138Y, R190S, K417T, E484K, N501Y, D614G, H655Y, T1027I | 8.44E-09 | 2.88E+05 | 2.43E-03 | 0.99 |
| S-Delta | T19R, G142D, EF156-157del, R158G, L452R, T478K, D614G, P681R, D950N | / | / | / | / |

**References**

1 Wrapp, D. et al. Cryo-EM structure of the 2019-nCoV spike in the prefusion conformation. *Science* **367**, 1260-1263 (2020).

2 Ying, T. et al. Exceptionally potent neutralization of Middle East respiratory syndrome coronavirus by human monoclonal antibodies. *J Virol.* **88**, 7796-7805 (2014).

3 Ying, T., Chen, W., Gong, R., Feng, Y. & Dimitrov, D. S. Soluble monomeric IgG1 Fc. *The Journal of biological chemistry.* **287**, 19399-19408 (2012).

4 Wang, Q. S. et al. The macromolecular crystallography beamline of SSRF. *Nuclear Science and Techniques.* **26**, 12-17 (2015).

5 Wang, Q.-S. et al. Upgrade of macromolecular crystallography beamline BL17U1 at SSRF. *Nuclear Science and Techniques.* **29**, 68 (2018).

6 Otwinowski, Z. & Minor, W. Processing of X-ray diffraction data. *Methods enzymol.* **276**, 307-326 (1997).

7 Vagin, A. & Teplyakov, A. MOLREP: an automated program for molecular replacement. *J Appl Crystallogr.* **30**, 1022-1025 (1997).

8 Vagin, A. A. et al. REFMAC5 dictionary: organization of prior chemical knowledge and guidelines for its use. *Acta Crystallogr D.* **60**, 2184-2195 (2004).

9 Adams, P. D. et al. PHENIX: a comprehensive Python-based system for macromolecular structure solution. *Acta crystallographica. Section D, Biological crystallography.* **66**, 213-221 (2010).

10 Emsley, P., Lohkamp, B., Scott, W. G. & Cowtan, K. Features and development of Coot. *Acta Crystallogr D.* **66**, 486-501 (2010).

11 DeLano, W. L. & Lam, J. W. PyMOL: A communications tool for computational models. *Abstr Pap Am Chem S* **230**, U1371-U1372 (2005).

12 Lu, L. et al. Structure-based discovery of Middle East respiratory syndrome coronavirus fusion inhibitor. *Nat Commun,* **5**, 3067 (2014).

13 Xia, S. et al. Inhibition of SARS-CoV-2 (previously 2019-nCoV) infection by a highly potent pan-coronavirus fusion inhibitor targeting its spike protein that harbors a high capacity to mediate membrane fusion. *Cell Res.* **30**, 343-355 (2020).

14 Yao, H. et al. Rational development of a human antibody cocktail that deploys multiple functions to confer Pan-SARS-CoVs protection. *Cell Res.* **31**, 25-36 (2021).

15 Li, S. et al. Mechanism of intracellular cAMP sensor Epac2 activation: cAMP-induced conformational changes identified by amide hydrogen/deuterium exchange mass spectrometry (DXMS). *J Biol Chem*. **286**, 17889-17897 (2011).
